# The secreted protein FonCHRD is essential for vegetative growth, asexual reproduction, and pathogenicity in watermelon Fusarium wilt fungus

**DOI:** 10.1007/s44297-024-00036-x

**Published:** 2024-10-25

**Authors:** Jiajun Lou, Jiajing Wang, Shanshan Zeng, Xiaohui Xiong, Mengmeng Guo, Dayong Li, Fengming Song

**Affiliations:** 1 Key Laboratory of Crop Diseases and Insect Pests of Ministry of Agriculture and Rural Affairs, Institute of Biotechnology, College of Agriculture and Biotechnology, Zhejiang University, Hangzhou, 310058 People’s Republic of China; 2 Zhejiang Provincial Key Laboratory of Biology of Crop Pathogens and Insects, Institute of Biotechnology, College of Agriculture and Biotechnology, Zhejiang University, Hangzhou, 310058 People’s Republic of China; 3 State Key Laboratory of Rice Biology and Breeding, Institute of Biotechnology, College of Agriculture and Biotechnology, Zhejiang University, Hangzhou, 310058 People’s Republic of China

**Keywords:** FonCHRD, *Fusarium oxysporum* f. sp. *niveum*, Pathogenicity, plant cell death, Secreted protein, Watermelon Fusarium wilt

## Abstract

Fungal pathogens often secrete numerous effectors to interfere with and/or suppress plant immunity to promote their infection. Watermelon Fusarium wilt, caused by the soil-borne fungus *Fusarium oxysporum* f. sp. *niveum* (*Fon*), is one of the devastating diseases that severely affect the watermelon industry. Here, we report the function of a candidate effector protein, FonCHRD, in *Fon*. FonCHRD harbors a chordin (CHRD) domain of unknown function and has a signal peptide with secretion activity. *FonCHRD* shows a relatively high expression level in *Fon* marcoconidia and is inducible by watermelon root tissues. Phenotypic analysis of the targeted deletion mutant revealed that FonCHRD plays roles in vegetative growth, asexual reproduction, and conidial morphology of *Fon*, while it is not involved in spore germination as well as cell wall, oxidative and salt stress responses. Deletion of *FonCHRD* impaired the ability to colonize and spread within host plants, significantly reducing its virulence on watermelon. FonCHRD is distributed across multiple compartments of plant cells but can target to the apoplast space in plants. FonCHRD inhibits the INF1- and Bcl2-associated X protein-triggered cell death and defense gene expression in transiently expressed *Nicotiana benthamiana* leaves. These findings suggest that FonCHRD is essential for *Fon* pathogenicity by modulating invasive growth and spreading abilities as well as by suppressing plant immune responses.

**Supplementary Information:**

The online version contains supplementary material available at 10.1007/s44297-024-00036-x.

## Introduction

*Fusarium oxysporum* can infect more than 150 plant species, including economically important crops such as cotton, tomatoes, melons, and bananas, resulting in great yield reductions and economic losses [1–4]. Watermelon Fusarium wilt, caused by *F. oxysporum* f. sp. *niveum* (*Fon*), is a devastating vascular disease that threatens the global watermelon industry [5–7]. Long-term survival in soil and emergence of new races of *Fon*, commercialization of susceptible cultivars, and lack of effective control measures make it difficult to manage this disease in the field [5–7].

*F. oxysporum* exhibits a hemibiotrophic lifestyle, with its initial biotrophic phase primarily occurring within plant root cortex cells, followed by invasion of the vascular system and transition to the necrotrophic phase [2]. Its colonization in the vascular system disrupts water transport in plants, leading to wilting symptoms and the death of infected plants [8, 9]. To successfully colonize the host plants, *F. oxysporum* produces a large number of virulence factors [10], including cell wall degrading enzymes such as pectinase, pectin lyase, xylanase, cellulase and hemicellulase [11] and toxins such as fumonisin, beauvericin, and fusaric acid [12]. Most importantly, *F. oxysporum* secretes a multitude of effector proteins, which serve as critical virulence factors and are employed to manipulate the plant immune system, either to facilitate infection or elicit a plant defense response [13].

The effector proteins can be roughly classified into apoplastic and cytoplasmic effectors according to their subcellular localization [14]. The Secreted In Xylem (SIX) effector proteins, were identified in the xylem sap of tomato plants infected with *F. oxysporum* f. sp. *lycopersici* (*Fol*) [15] and many of them have been shown to play pivotal roles in the *F. oxysporum*-plant interactions [16, 17], either as suppressors of plant pattern-triggered immunity [18] or as determinant factors for the pathogenicity and host specificity of the pathogen [19, 20]. Recently, many candidate effectors have been identified in the *F. oxysporum*, especially in *Fol*, *F. oxysporum* f. sp. *cubense* (*Foc*), and the *F. oxysporum* isolate Fo5176 based on their genome sequence information [10] and extensive functional studies have revealed diverse functions and distinct mechanisms of the effector proteins in manipulating the plant immune system. FolAsp, which is necessary for *Fol* full virulence, inhibits plant defense mechanisms, including ROS burst, through hydrolyzing plant proteins via its protease activity [21]. Serine protease FoSep1 and metalloprotease FoMep1 of *Fol* and the metalloprotease FocM35_1 of *Foc* suppress plant chitinase activity to protect the fungal cell wall integrity [22, 23]. *Fol*-secreted FolSvp1 and *Foc*-secreted FocCP1 directly interacts with tomato and banana pathogenesis-related proteins PR1s to affect their subcellular localization and antifungal activity, respectively, thereby promoting infection [24, 25]. FoRnt2 and FoAPY1 are involved in *Fol* virulence through their RNase and peptidase activities, respectively [26, 27]. Furthermore, many effector proteins, such as FoCupin1, Fol-EC14, Fol-EC20, and FoSSP17, can suppress plant cell death in *Nicotiana benthamiana* and are required for fungal pathogenicity [28–30]. However, the mechanisms by which the effector proteins contribute to the pathogenicity of *F. oxysporum* remain largely unknown.

Chordin (CHRD) is a secreted protein initially isolated from the Spemann organizer of *Xenopus gastrula* [31, 32] and its transcription is precisely regulated by homeobox transcription factors during neuroectoderm and mesoderm germ-layer specification [33, 34]. In vertebrates, the function of the *CHRD* gene has been extensively studied; for instance, CHRD acts as an antagonist of bone morphogenetic proteins, thereby exerting crucial roles in numerous important developmental processes [35–39]. The amplification of *CHRD* in a variety of human cancer cells makes it potentially valuable as a cancer biomarker for clinical applications [40]. In green algae (*Chlamydomonas* W80), CHRD domain-containing secreted proteins are associated with stress responses [41]. The CHRD domain-containing proteins are also present in many microbial proteins [42]. In *Fol* genome, a protein containing CHRD domain was identified as a secreted effector protein with a high likelihood of involvement in pathogenicity [43]. However, the function of the CHRD domain-containing effector proteins in fungal pathogenicity is still elusive.

In this study, we investigated the function of a candidate effector protein with CHRD domain, namely as FonCHRD, in *Fon*. Results from phenotypic analysis of the targeted deletion mutant revealed that FonCHRD is essential for vegetative growth, asexual reproduction, and pathogenicity of *Fon*. Moreover, FonCHRD can inhibit the INF1- and Bcl2-associated X protein (BAX)-induced immune responses in *N. benthamiana* leaves. These findings demonstrate that FonCHRD play critical roles in *Fon* pathogenicity through affecting colonization and spreading abilities within watermelon plants and interfering with plant immune responses.

## Results

### *FonCHRD* encodes a secreted protein and responds to watermelon roots

**Fig. 1:**
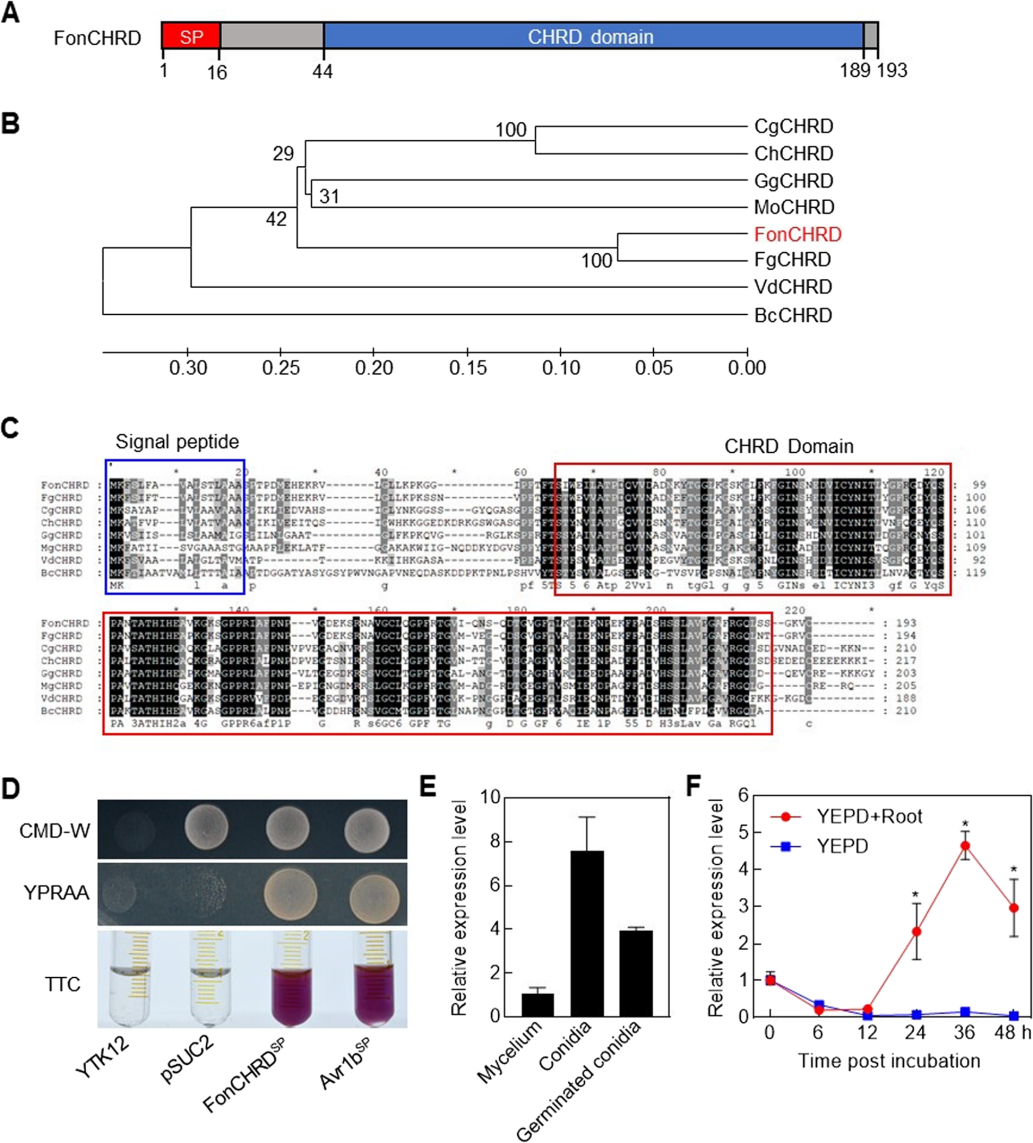
*FonCHRD* encodes a CHRD domain-containing secreted protein and responds to watermelon root. **A** Structure of FonCHRD protein. SP, signal peptide. **B** Phylogenetic relationships of FonCHRD with CHRD proteins from other fungi. **C** Sequence alignment of FonCHRD with CHRD proteins from other fungi. Signal peptide and CHRD domain are indicated with blue and red boxes, respectively. Fon, *Fusarium oxysporum* f. sp. *niveum*; Fg, *Fusarium graminearum*; Bc: *Botrytis cinerea*; Mo, *Magnaporthe oryzae*; Gg, *Gaeumannomyces graminis* var. *tritici*; Cg, *Colletotrichum graminicola*; Ch, *Colletotrichum higginsianum*; Vd, *Verticillium dahliae*. **D** Secretion activity of the signal peptide in FonCHRD. Yeast YTK12 strains carrying different plasmids were grown on CMD-W or YPRAA medium for 3 d. Secretion activity was determined by the growth performance on YPRAA medium and the appearance of red insoluble 1,3,5-triphenylformic acid after the addition of 2,3,5-triphenyltetrazolium chloride (TTC) in the cultures. **E** Expression patterns of *FonCHRD* in macroconidia, germinated macroconidia, and mycelia of *Fon*. **F** Induction of *FonCHRD* expression by watermelon roots. Transcript level of *FonCHRD* in *Fon* macroconidia with or without watermelon root induction at the indicated time points was measured by RT-qPCR. *FonActin* was used as an internal reference. The experiments were independently conducted three times and results from one representative experiment are shown in (**D**). Data presented in (**E**) and (**F**) are means ± SD from three independent experiments and asterisks indicate significant differences (*p* < 0.05, Student’s *t* test)

FOXG_08152 was predicted to encode a candidate effector protein with a CHRD domain in *Fol* [43]. In our preliminary experiments, *Fon* strains with deletions in several predicted effector proteins, including FonCHRD, exhibited reduced pathogenicity on watermelon plants, Therefore, we focused on the function of FonCHRD in *Fon*. The gene corresponding to FOXG_08152 was amplified from *Fon* and named as *FonCHRD* (Fig. S1A). *FonCHRD* encodes a 193 amino acid (aa) protein, containing a CHRD domain (Fig. 1A). FonCHRD is highly enriched in random coil, accounting for 59.6% (Fig. S1B) and its three-dimensional structure shows 86.5% of similarity to the remodeled proteins with the CHRD domain in the database (Fig. S1B, C). CHRD domain-containing proteins were also identified in several important plant pathogenic fungi, including *Magnaporthe oryzae*, *Colletotrichum* spp., *Fusarium* spp., *Botrytis cinerea*, *Verticillium dahliae*, and *Gaeumannomyces graminis* var. *tritici*. FonCHRD was closely related to FgCHRD from *F. graminearum* but had a more distant evolutionary relationship with CHRD proteins from *B. cinerea* and *V. dahlia* (Fig. 1B). The CHRD domain-containing proteins from various plant pathogenic fungi are highly conserved, particularly within the CHRD domain (Fig. 1C), implying the conservative roles of these proteins in pathogenic fungi. FonCHRD contains an N-terminal signal peptide (SP; 16 amino acids) (Fig. 1C) but lacks transmembrane domains and glycosylphosphatidylinositol (GPI) anchor sites (Fig. S1D), suggesting its secretion into the extracellular space. FonCHRD is classified as a conventional secretory protein and is likely to function as an apoplastic/cytoplasmic effector, with a probability of > 0.5 (Fig. S1D).

A yeast SP screen trap assay, in which yeast strains can grow on yeast extract-peptone-raffinose-antimycin A-agar (YPRAA) medium only when invertase is secreted [44] was conducted to verify the secretory activity of the SP in FonCHRD. The results showed that, similar to the YTK12 strain carrying pSUC2-Avr1b^SP^ [45], the YTK12 strain carrying pSUC2-FonCHRD^SP^ was able to grow on YPRAA medium and reduced the 2,3,5-triphenyltetrazolium chloride to the red insoluble 1,3,5-triphenylformic (Fig. 1D), indicating that the SP in FonCHRD is functional for secretion.

To gain a preliminary insight into the function of *FonCHRD*, we first analyzed its expression patterns at different developmental stages of *Fon* and its responsiveness to host plant induction. *FonCHRD* exhibited the highest expression level in macroconidia, followed by the level in germinating spores for 12 h, with the lowest level in mature hyphae of *Fon* (Fig. 1E). Upon addition of watermelon roots to the spore germinating medium, the expression level of *FonCHRD* began to increase at 24 h and peaked at 36 h post incubation, leading to an increase of ~ 30 folds compared to that in the medium without watermelon roots (Fig. 1F). These results indicate that *FonCHRD* is predominantly expressed in macroconidia of *Fon* and responds effectively to watermelon roots, implying a possible involvement in pathogenesis on watermelon plants.

### *FonCHRD *is essential for the pathogenicity of *Fon*

**Fig. 2:**
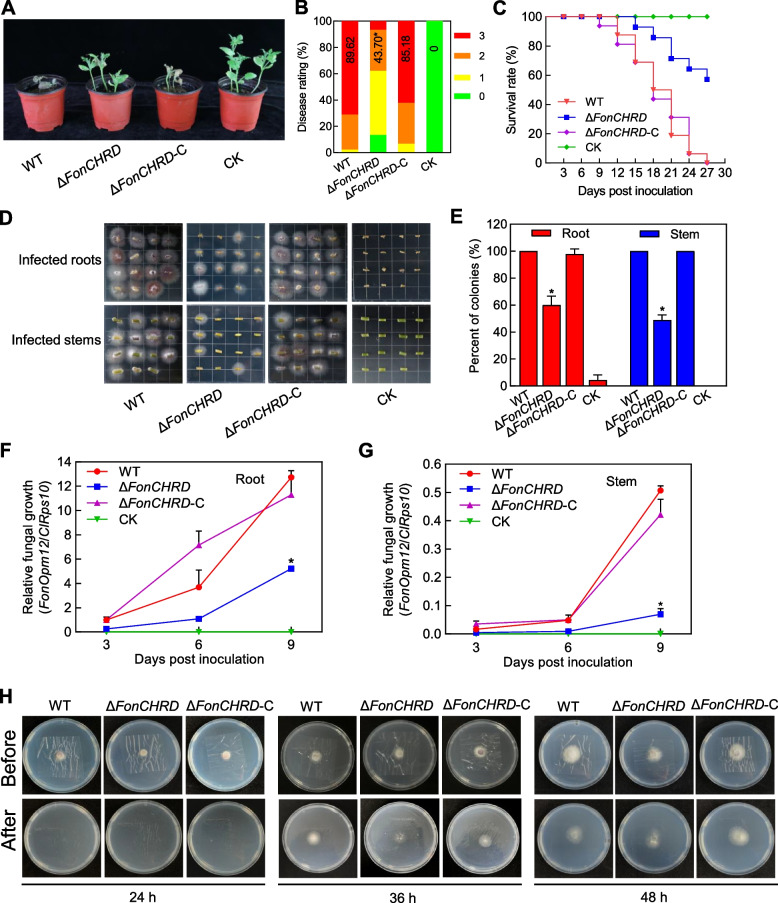
*FonCHRD* is essential for the pathogenicity of *Fusarium oxysporum* f. sp. *niveum*. **A**–**C** Disease phenotype (**A**), disease index (**B**), and survival curves (**C**) of watermelon plants after inoculation with Δ*FonCHRD*, Δ*FonCHRD*-C, or WT strains. Photographs were taken at 21 dpi. **D**–**E** Growth phenotypes (**D**) and percentages (**E**) of fungal colonies recovered from roots and stems of the Δ*FonCHRD*-, Δ*FonCHRD*-C-, or WT-inoculated watermelon plants at 15 dpi. The sampled roots and stems of the infected plants were sterilized with 75% ethanol and incubated on PDA plates for 3 d. **F** and **G** *In planta* fungal biomass in roots (**F**) and stems (**G**) of the Δ*FonCHRD*-, Δ*FonCHRD*-C-, or WT-inoculated watermelon plants. Relative fungal biomass was analyzed by qPCR and calculated as ratios of *FonOpm12/ClRps10*. **H** Penetration ability of Δ*FonCHRD*, Δ*FonCHRD*-C and WT against cellophane membranes. The tested strains were inoculated on cellophane membranes on MM plates for indicated times, and the cellophane membranes were removed and grown for another 2 d to observe the mycelial growth. The experiments in (**A**), (**C**), (**D**) and (**H**) were independently conducted three times with similar results and data presented in (**B**), (**E**), (**F**) and (**G**) are means ± SD from three independent experiments. Asterisks indicate significant differences (*p* < 0.05, Student’s *t* test)

With the generated targeted deletion mutant Δ*FonCHRD-20* (Fig. S2A–C) and its complementation strain Δ*FonCHRD*-C (Fig. S2D), we investigated whether targeted disruption of *FonCHRD* affected *Fon* pathogenicity on watermelon. Given the similar phenotypes of the three mutant lines Δ*FonCHRD-7*, Δ*FonCHRD-11*, and Δ*FonCHRD-20* showing decreased pathogenicity on watermelon plants in preliminary experiments (Fig. S3), we selected Δ*FonCHRD-20* for further study. The overall disease symptoms in Δ*FonCHRD*-inoculated plants were significantly relieved in contrast with those in the wild-type (WT)-inoculated plants (Fig. 2A). Specifically, only 7% of the Δ*FonCHRD*-inoculated plants died, with 49% showing leaves chlorosis and 13% without symptoms, whereas 71% of the WT-inoculated plants exhibited severe wilting and death at 21 days post inoculation (dpi), resulting in a 51% decrease in disease severity compared to the WT-inoculated plants (Fig. 2B). The Δ*FonCHRD*-C strain restored the pathogenicity defect in Δ*FonCHRD*, as the Δ*FonCHRD*-C-inoculated plants showed comparable disease symptoms and severity to the WT-inoculated plants (Fig. 2A and B), confirming that the pathogenicity defect in Δ*FonCHRD* is due to the deletion of *FonCHRD*. Furthermore, the dynamic survival curves revealed that both WT- and Δ*FonCHRD*-C-inoculated plants started dying at 9–12 dpi, while the Δ*FonCHRD*-inoculated plants began dying at 15 dpi, leading to a 3 ~ 6 d delay. At 27 dpi, all the WT and Δ*FonCHRD*-C-inoculated plants died, while only 43% of the Δ*FonCHRD*-inoculated plants were dead (Fig. 2C). These results indicate that *FonCHRD* is essential for *Fon* pathogenicity.

To elucidate the possible mechanism by which *FonCHRD* affects *Fon* pathogenicity, we compared the invasion growth and penetration ability of the Δ*FonCHRD*, Δ*FonCHRD*-C, and WT strains. In the tissue isolation experiments, typical *Fon* colonies appeared on 60% of roots and 49% of stems collected from the Δ*FonCHRD*-inoculated plants at 15 dpi, which were significantly lower than the percentage of *Fon* colonies (96% ~ 100%) detected in the roots and stems from the WT and Δ*FonCHRD*-C-inoculated plants (Fig. 2D and E). Furthermore, relative *in planta* fungal biomass was significantly reduced in roots and stems of the Δ*FonCHRD*-inoculated plants compared to those of the WT- and Δ*FonCHRD*-C-inoculated plants at 6 and 9 dpi, leading to decreases of 56% in roots and 85% in stems (Fig. 2F and G). Notably, the reduction of Δ*FonCHRD* biomass in stems was much more evident than that in roots (Fig. 2G). Surprisingly, the Δ*FonCHRD* strain exhibited similar penetration ability against cellophane membrane to that of the WT and Δ*FonCHRD*-C strains after incubation on the membranes for 36 h and 48 h (Fig. 2H). These results suggest that *FonCHRD* regulates *Fon* pathogenicity largely through affecting its colonization and spreading within watermelon plants rather than its penetration ability.

### *FonCHRD* is required for mycelial growth, asexual reproduction, and conidial morphology of *Fon*

**Fig. 3:**
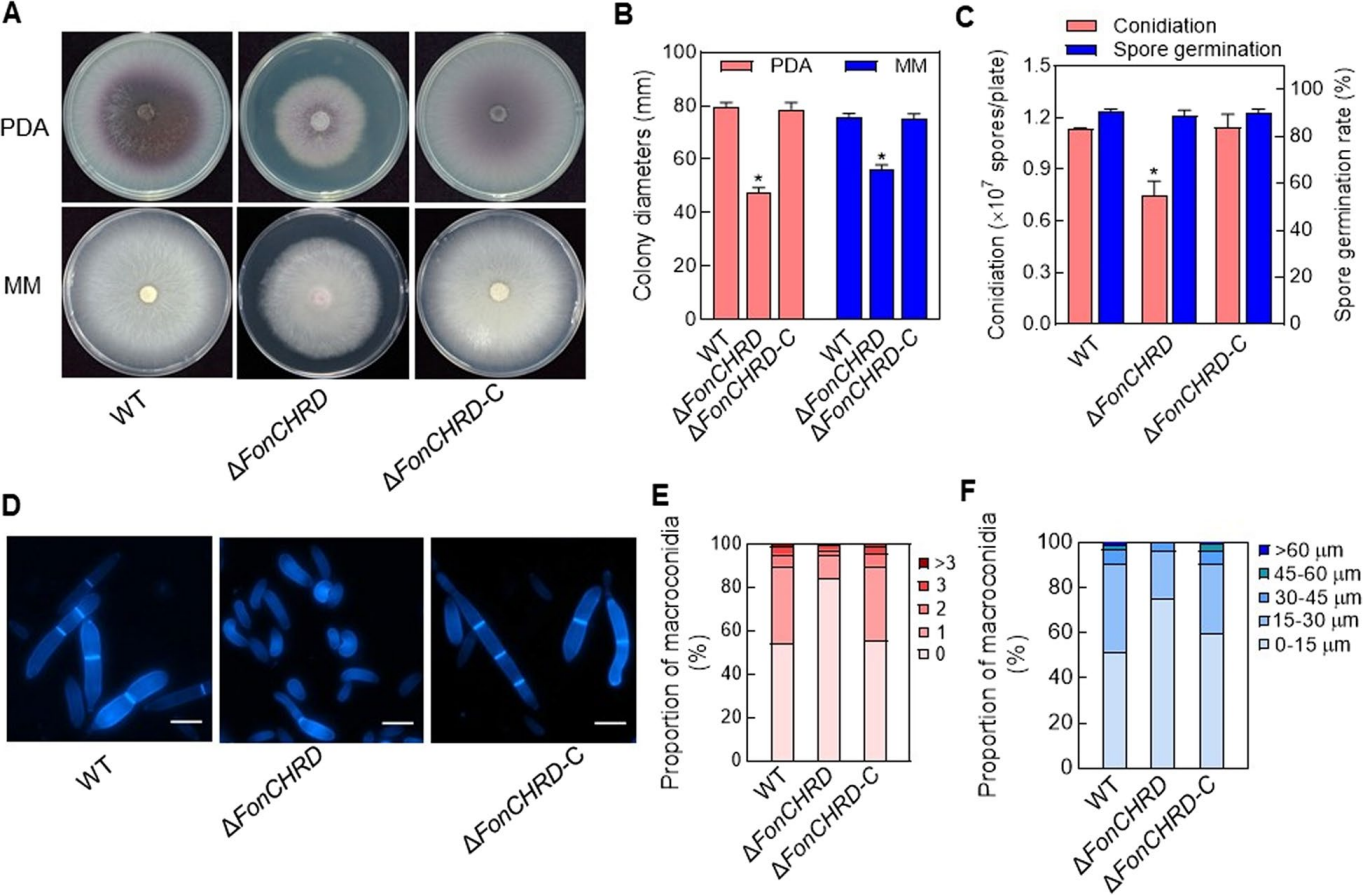
Functions of *FonCHRD* in basic biological processes of *Fusarium oxysporum* f. sp. *niveum*. **A** and** B** Mycelial growth (**A**) and colony diameters (**B**) of the Δ*FonCHRD*, Δ*FonCHRD*-C, and WT strains on PAD and MM plates. **C** Conidiation and spore germination of the Δ*FonCHRD*, Δ*FonCHRD*-C, and WT strains. Macroconidia numbers were counted after incubating the tested strains in mung bean broth for 2 d at 200 rpm, 26℃. Germination rates of indicated strains were estimated after suspending their macroconidia in YEPD broth and incubating for 12 h at 200 rpm, 26℃. **D** Morphology of macroconidia produced by the Δ*FonCHRD*, Δ*FonCHRD*-C, and WT strains. Macroconidia were observed after staining with Calcofluor White for 5 min. Scale bar = 10 μm. **E** and **F** Proportions of the Δ*FonCHRD*-, Δ*FonCHRD*-C-, and WT-produced macroconidia with different septum number (**E**) and length (**F**). The experiments were preformed independently three times with similar results and results from one representative experiment are shown in (**A**) and (**D**). Data presented in (**B**), (**C**), (**E**) and (**F**) are means ± SD from three independent experiments and asterisks indicate significant differences (*p* < 0.05, Student’s *t* test)

We further examined the potential involvement of *FonCHRD* in the basic biological processes of *Fon* by comparing the mycelial growth, conidiation, spore germination and macroconidial morphology of the Δ*FonCHRD*, Δ*FonCHRD*-C and WT strains. When grown on potato dextrose agar medium (PDA) or minimal medium (MM), Δ*FonCHRD* exhibited significantly slower mycelial growth compared to WT and Δ*FonCHRD*-C (Fig. 3A), resulting in reductions of 44% on PDA and 26% on MM in colony diameters (Fig. 3B). After a 48-h incubation in mung bean liquid (MBL) medium, Δ*FonCHRD* produced fewer macroconidia than WT and Δ*FonCHRD*-C, leading to a 34% reduction (Fig. 3C); however, the germination rate of the macroconidia produced by Δ*FonCHRD* was comparable to those of WT and Δ*FonCHRD*-C (Fig. 3C). Moreover, the macroconidia produced by Δ*FonCHRD* exhibited abnormal morphology, e.g., irregular curling, compared to those of WT and Δ*FonCHRD*-C (Fig. 3D). The Δ*FonCHRD*-produced macroconidia had fewer septa with shorter length compared to those generated by WT and Δ*FonCHRD*-C (Fig. 3E and F). These results indicate that *FonCHRD* is involved in regulating vegetative growth, asexual reproduction, and macroconidial morphology but not in the germination of macroconidia in *Fon*.

### *FonCHRD* is not involved in cell wall, oxidative and salt stress responses of *Fon*

**Fig. 4:**
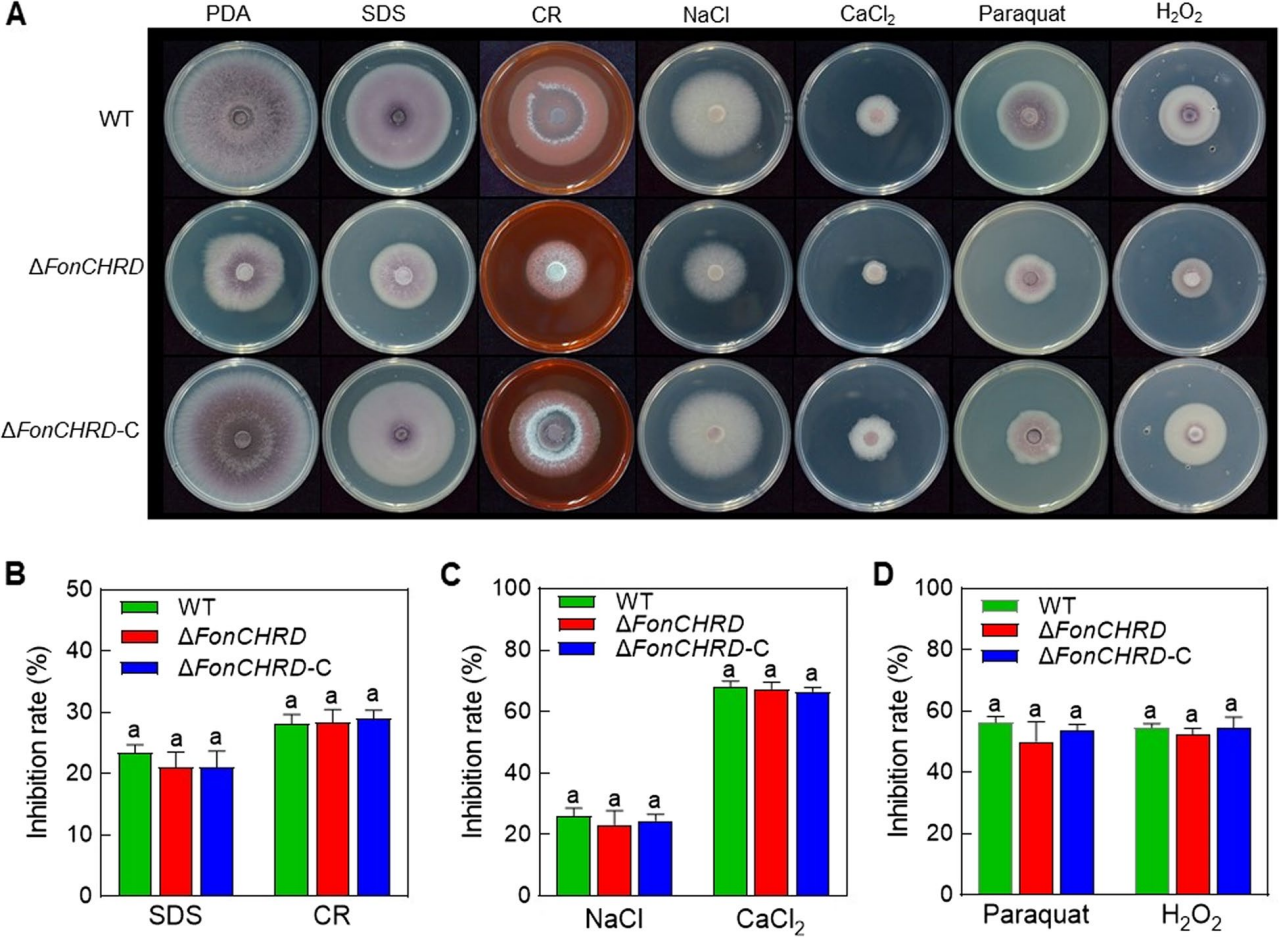
*FonCHRD* is not involved in cell wall, oxidative and salt stress response of *Fusarium oxysporum* f. sp. *niveum*. **A** Mycelial growth of Δ*FonCHRD*, Δ*FonCHRD*-C, and WT on PDA supplemented with different abiotic stress agents at 7 d. **B**-**D** Growth inhibition rates of Δ*FonCHRD*, Δ*FonCHRD*-C, and WT on PDA plates under cell wall stress (**B**), salt stress (**C**), and oxidative stress (**D**). The experiments were performed independently three times with similar results. Data are shown as means ± SD from three independent experiments and the same letters indicate no significant difference among the strains under the same stress condition (*p* < 0.05, one-way ANOVA)

We also examined the involvement of *FonCHRD* in abiotic stress responses of *Fon* by comparing the mycelial growth inhibition rates of Δ*FonCHRD*, Δ*FonCHRD*-C, and WT on PDA plates amended with various stress-inducing agents. No significant difference was detected in the inhibition rates of mycelial growth of Δ*FonCHRD* compared to those of WT and Δ*FonCHRD*-C when grown on PDA plates containing cell wall stress agents, e.g., sodium dodecyl sulfate (SDS) and Congo Red (CR), oxidative stress agents,e.g., H_2_O_2_ and paraquat, and salt stress chemicals, e.g., NaCl and CaCl_2_ (Fig. 4A–D). These results suggest that *FonCHRD* does not participate in cell wall, oxidative and salt stress responses of *Fon*.

### FonCHRD targets to apoplast space of plants

**Fig. 5:**
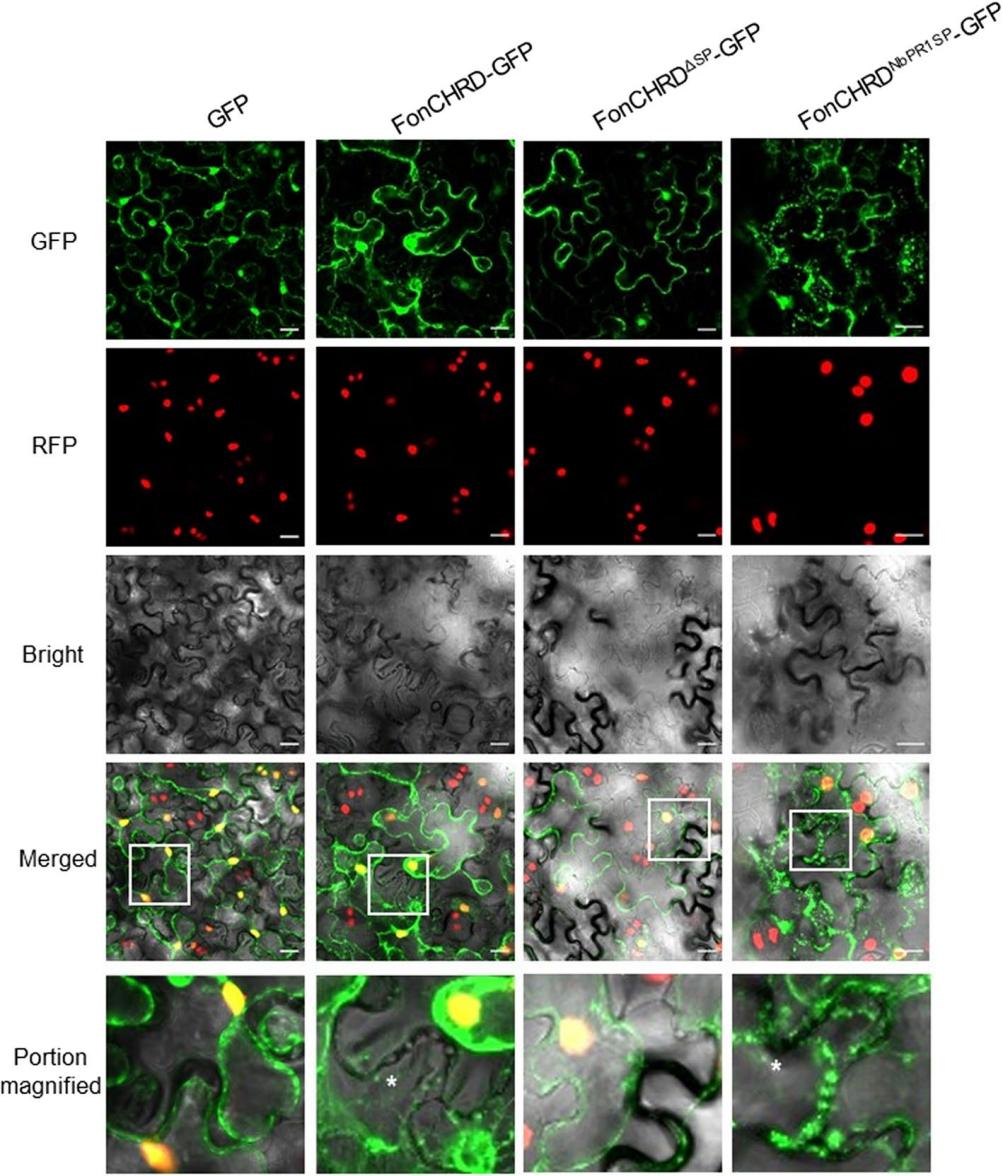
FonCHRD can be secreted into apoplast space in transiently expressed *Nicotiana benthamiana* leaves. FonCHRD-GFP, FonCHRD^ΔSP^-GFP and FonCHRD^NbPR1SP^-GFP were transiently expressed in *N. benthamiana* leaves and the agroinfiltrated leaves were treated with 0.8 M mannitol for plasmolysis, followed by observations under a confocal microscopy. Image areas selected by the white boxes are magnified and asterisks indicate the localization of the observed green fluorescence in apoplast spaces. Scale bars = 20 μm. The experiments were independently performed three times with similar results and data from one representative experiment are shown

To validate the subcellular localization of FonCHRD in plants, we transiently expressed a GFP-tagged intact FonCHRD, a GFP-tagged SP-deleted variant FonCHRD^ΔSP^, and a GFP-tagged hybrid variant FonCHRD^NbPR1SP^, in which the native SP was replaced with NbPR1 SP, in *N. benthamiana* leaves. The fusion proteins of FonCHRD-GFP, FonCHRD^ΔSP^-GFP, and FonCHRD^NbPR1SP^-GFP were detected in transiently expressed *N. benthamiana* leaves in Western blot analysis (Fig. S4A). Confocal microscopic observations showed that FonCHRD-GFP was localized in the cytoplasm, nucleus, and plasma membrane, while FonCHRD^ΔSP^-GFP was found in the nucleus and plasma membrane (Fig. S4B). FonCHRD^NbPR1SP^-GFP was specifically localized around the plasma membrane (Fig. S4B). Notably, both FonCHRD-GFP and FonCHRD^NbPR1SP^-GFP aggregated as puncta in the cytoplasm or near the plasma membrane (Fig. S4B). To clarify whether FonCHRD is secreted into the apoplast space, we further examined the subcellular localization of FonCHRD-GFP, FonCHRD^ΔSP^-GFP, and FonCHRD^NbPR1SP^-GFP in transiently expressed *N. benthamiana* leaves after treatment with 0.8 M mannitol for plasmolysis. The results showed that FonCHRD-GFP and FonCHRD^NbPR1SP^-GFP were present in the apoplast space, whereas FonCHRD^ΔSP^-GFP was absent (Fig. 5). These data indicate that FonCHRD can be secreted into the apoplast space of plants and that SP directs this process, which is consistent with the subcellular localization of FonCHRD predicted by EffectorP 3.0 (Fig. S1D).

### FonCHRD inhibits INF1- and BAX-induced cell death in *N. benthamiana* leaves

**Fig. 6:**
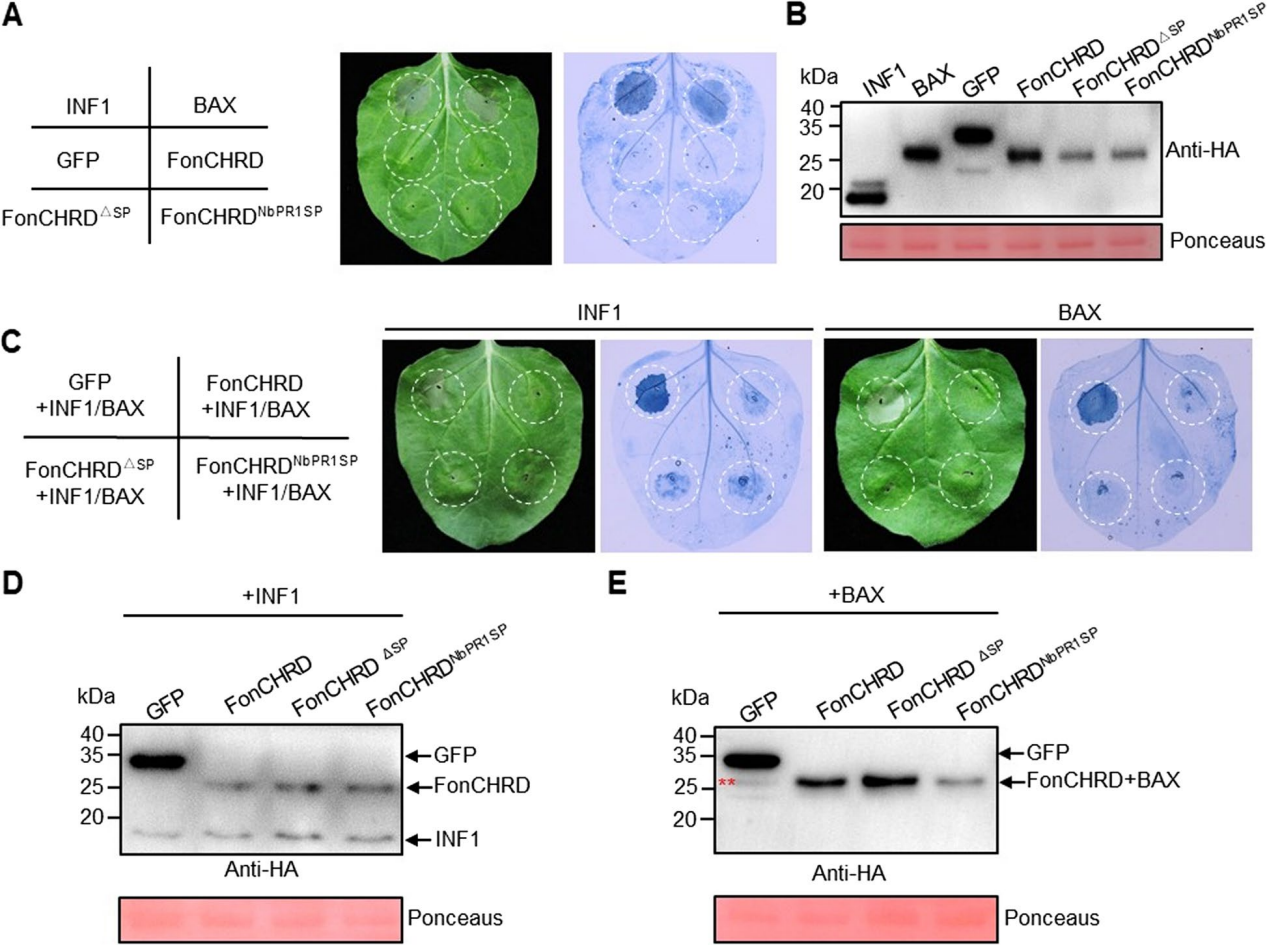
FonCHRD inhibits INF1- and BAX-induced cell death in transiently expressed *Nicotiana benthamiana* leaves. **A** Experimental design (*left*), necrotic phenotype (*middle*) and Trypan blue-stained dead cells (*right*) in *N. benthamiana* leaves transiently expressing FonCHRD-HA, FonCHRD^ΔSP^-HA or FonCHRD^NbPR1SP^-HA. INF1 and BAX were used as positive controls while GFP was served as a negative control. Photographs were taken at 4 d post-agroinfiltration. **B** Western blot detection of FonCHRD-HA, FonCHRD^ΔSP^-HA and FonCHRD^NbPR1SP^-HA in transiently expressed *N. benthamiana* leaves of (**A**). Ponceau S staining was used for equal loading of protein samples. **C** Experimental design (*left*), necrotic phenotype (*middle*) and Trypan blue-stained dead cells (*right*) in *N. benthamiana* leaves transiently co-expressing FonCHRD-HA, FonCHRD^ΔSP^-HA and FonCHRD^NbPR1SP^-HA with INF1 or BAX. Photographs were taken at 4 d post-agroinfiltration. GFP was used as a negative control. **D**–**E** Western blot detection of FonCHRD-HA, FonCHRD^ΔSP^-HA and FonCHRD^Nb^.^PR1SP^-HA with INF1 (**D**) or BAX (**E**) in transiently co-expressed *N. benthamiana* leaves of (**C**). Red double asterisk indicates the BAX band in (**E**). The experiments were independently performed three times with similar results and results from one representative experiment are shown in (**A**) and (**C**)

To investigate the impact of FonCHRD on plant immune responses related to its function in *Fon* pathogenicity, we examined the plant programmed cell death (PCD) phenotype in *N. benthamiana* leaves transiently expressing HA-tagged FonCHRD, FonCHRD^ΔSP^, or FonCHRD^NbPR1SP^. We used INF1 and BAX, known PCD inducers in *N. benthamiana* leaves [46, 47], as positive controls. We observed clear PCD in *N. benthamiana* leaves transiently expressing INF1 or BAX but did not see PCD in leaves transiently expressing FonCHRD-HA, FonCHRD^ΔSP^-HA, or HA-FonCHRD^NbPR1SP^-HA (Fig. 6A). Western blot results confirmed the presence of FonCHRD-HA, FonCHRD^ΔSP^-HA, and FonCHRD^NbPR1SP^-HA in *N. benthamiana* leaves (Fig. 6B), indicating that the absence of PCD in *N. benthamiana* leaves expressing FonCHRD-HA, FonCHRD^ΔSP^-HA, or FonCHRD^NbPR1SP^-HA was not due to misexpression of these fusion proteins. These results suggest that FonCHRD does not possess the ability to induce PCD in plants.

We then analyzed the ability of FonCHRD to suppress INF- and BAX-triggered PCD in *N. benthamiana* leaves, examining whether it inhibits plant immune responses. For this purpose, we transiently co-expressed FonCHRD-HA, FonCHRD^ΔSP^-HA, or FonCHRD^NbPR1SP^-HA in *N. benthamiana* leaves with INF1 or BAX. At 4 d post-agroinfiltration, both INF1 and BAX triggered obvious PCD in the GFP-expressed regions; however, this PCD disappeared or alleviated in *N. benthamiana* leaves transiently expressing FonCHRD-HA, FonCHRD^ΔSP^-HA or FonCHRD^NbPR1SP^-HA (Fig. 6C). The existence of FonCHRD-HA, FonCHRD^ΔSP^-HA, and FonCHRD^NbPR1SP^-HA as along with INF1 (Fig. 6D) and BAX (Fig. 6E) were confirmed in Western blot assays. Notably, BAX was indistinguishable in the FonCHRD-HA, FonCHRD^ΔSP^-HA, or FonCHRD^NbPR1SP^-HA bands owing to their similar sizes in Western blotting assays (Fig. 6E). Considering that FonCHRD^ΔSP^-HA, like FonCHRD-HA and FonCHRD^NbPR1SP^-HA, was also capable of suppressing the INF1- and BAX-triggered PCD in *N. benthamiana* leaves (Fig. 6C), these data suggest that FonCHRD inhibits INF1- and BAX-triggered PCD in *N. benthamiana* leaves, which is independent of its SP.

### FonCHRD inhibits the INF1- and BAX-induced expression of defense genes and chitin-triggered immune responses in *N. benthamiana* leaves

**Fig. 7:**
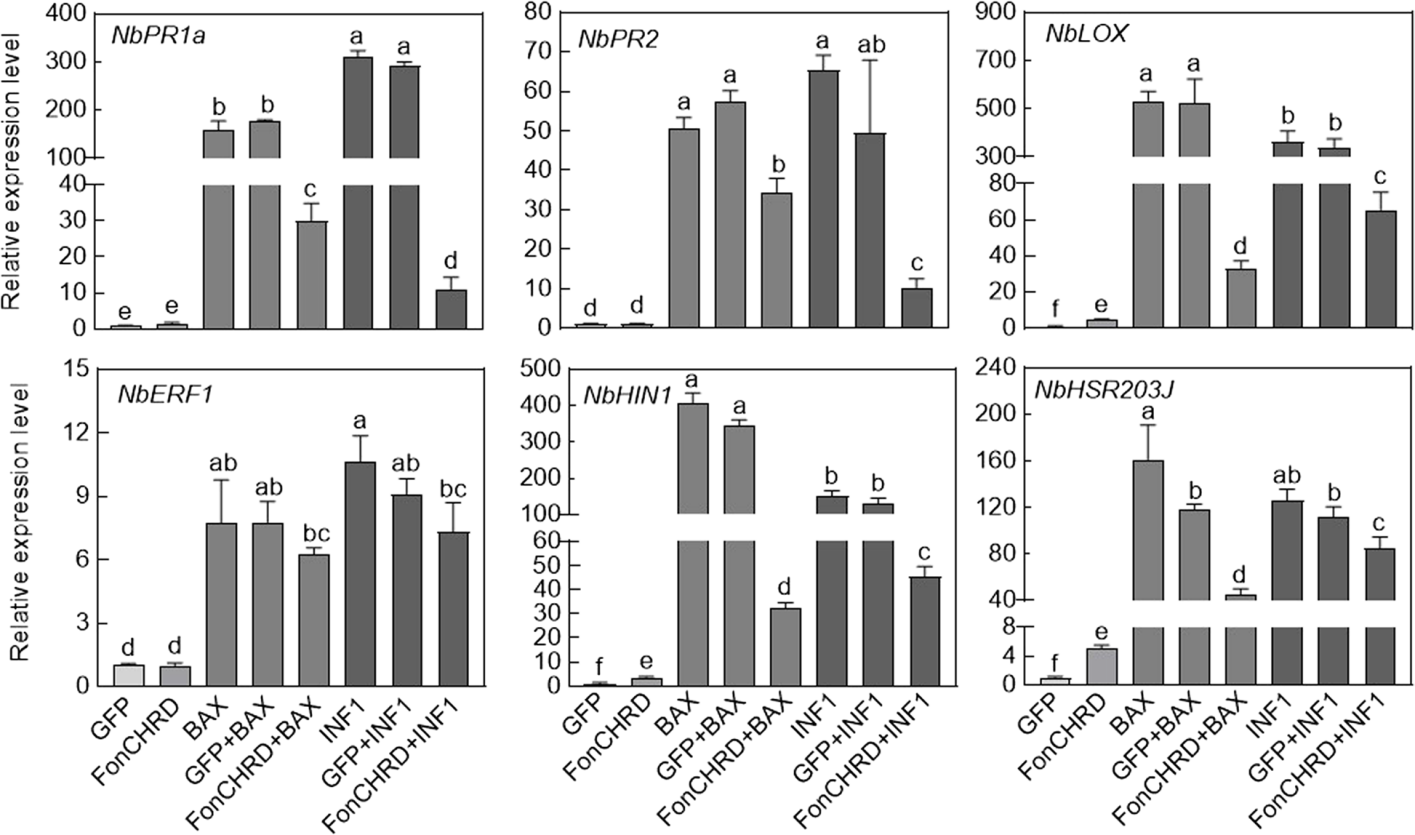
FonCHRD inhibits the INF1- and BAX-induced expression of defense genes in *Nicotiana benthamiana* leaves. Agrobacteria carrying GFP, FonCHRD-HA, BAX, or INF1 were infiltrated individually or in combinations into *N. benthamiana* leaves and leaf samples were collected for gene expression analysis at 48 h post-agroinfiltration. *NbGAPDH* was used as an internal reference. Data presented are means ± SD from three independent experiments and different letters indicate significant differences (*p* < 0.05, one-way ANOVA)

To further elucidate the suppression of plant immune responses by FonCHRD, we analyzed the expression changes of defense genes, including *NbPR1*, *NbPR2*, *NbLOX*, *NbERF1*, *NbHIN1*, and *NbHSR203J*, in *N. benthamiana* leaves after transient co-expression of FonCHRD-HA with INF1 or BAX [48]. FonCHRD -HA alone did not affect the expression of these defense genes; however, INF1/BAX, either alone or combining with GFP, significantly upregulated their expression levels (Fig. 7). Importantly, the expression levels of these defense genes were markedly decreased in* N. benthamiana* leaves transiently co-expressing FonCHRD-HA with INF1 or BAX compared to those in leaves expressing INF1, BAX, or in combination with GFP (Fig. 7). These results suggest that FonCHRD inhibits the INF1- or BAX-triggered expression of defense genes in *N. benthamiana*, implying that FonCHRD might be a facilitator of *Fon* infection through suppressing plant immune responses.

## Discussion

The secretory CHRD domain-containing proteins have been studied in chordates, green alga, and mammals, with roles in embryonic development in chordates [34] and abiotic stress responses in the halotolerant green alga [41]. The CHRD domain-containing protein is predicted to be a highly pathogenic candidate effector protein in *Fol* [43]; however, its function in plant pathogenic fungi remains elusive. In this study, we characterized FonCHRD as a potential effector in *Fon* and revealed that FonCHRD is critical for vegetative growth, asexual reproduction, and pathogenicity in *Fon*. Importantly, FonCHRD possesses the ability to inhibit INF1- or BAX-triggered PCD and upregulation of defense genes in *N. benthamiana* leaves. These findings uncover the function of the CHRD domain-containing proteins, like FonCHRD, in plant pathogenic fungi, especially in pathogenicity.

Secreted proteins, particularly effector proteins, are pivotal biochemical weapons employed by pathogens to evade or suppress host plant immunity for infection [49]. Advancements in next-generation sequencing, transcriptome profiling, bioinformatics tools, and machine learning have strongly facilitated the prediction and identification of secreted and effector proteins in *F. oxypsorum* [19, 43, 50–52]. Although the above-mentioned studies have yielded numerous candidate effectors in *F. oxypsorum*, the functional characterization of these effectors is still relatively limited [10]. Therefore, the precise identification and functional studies of candidate effectors are crucial for understanding the molecular mechanism of pathogenicity in *F. oxypsorum*. In plant pathogenic fungi, effector proteins typically contain N-terminal SP and are secreted via the conventional endoplasmic reticulum-Golgi pathway [53]. FonCHRD, harboring a SP with secretion activity (Fig. 1C and D), adheres to this traditional secretory pathway. Transcriptomic data analysis reveals that the upregulation of effector genes, induced by host plants during early stage of infection, is a common feature [28, 54, 55]. Our experiments revealed that *FonCHRD* is induced by root tissue of watermelon plants (Fig. 1F), suggesting that *FonCHRD* encodes a protein with effector characteristics.

Subcellular localization is a key point toward understanding the functions of effector proteins in the interactions of pathogens with their host plants. Apoplastic effectors, which target the extracellular space of host plant cells, can interfere with and counter plant-produced proteases, protease inhibitors, secondary metabolites, hydrolases, and plant antimicrobial compounds, thereby playing crucial roles in protecting pathogens and evading plant immune recognition [14, 56]. For instance, *Fol* secretes the aspartate family protein FolAsp to the apoplast space of plant cells, where it degrades defense-related proteins through its protease activity, suppressing ROS bursts and other immune responses [21]. Cytoplasmic effectors, which are secreted by pathogens, typically target subcellular components in plant cells, suppressing upstream immune responses or reprogramming host transcription, thereby facilitating pathogen infection. For example, FoRnt2 operates by degrading plant RNA, disrupting normal plant functions and aiding infection [27]. EffectorP 3.0, a machine learning-based software for predicting fungal candidate effectors [50], predicts FonCHRD as an apoplastic/cytoplasmic effector (Fig. S1D). Like most of the current studies exploring the subcellular localization of effector proteins through heterologous expression in plants, FonCHRD was transiently expressed in *N. benthamiana* leaves and found to be distributed in multiple subcellular compartments of cells (Fig S4). Importantly, FonCHRD can be secreted to apoplast space, which is directed by its SP (Fig. 5), implying that FonCHRD may exert its biochemical functions in the apoplast space. Intriguingly, FonCHRD formed spots in cytoplasmic compartment of the cells transiently expressing the protein (Fig. 5). Similar observations have been reported for other effectors or secreted proteins in *F. oxysporum*, e.g., FoSSP17 and Foa3 [30, 57], yet the significance and underlying mechanism of this phenomenon remain unknown. Different subcellular localizations of FonCHRD in plants may satisfy its multiple functions in difference biological processes including pathogenicity, vegetative growth, and asexual reproduction (Figs. 2 and 3). The unidentified biochemical activity of FonCHRD is also an obstacle to explore its exact subcellular localization in plants. Further investigations on the subcellular localization of FonCHRD in watermelon plants during infection process will clarify this key issue. Notably, FonCHRD exhibits a nuclear localization (Fig. S4). Generally, nucleus-localized effectors can interfere with and suppress plant immune responses, such as the interaction of nuclear protein FSE1 in *Foc* with banana MYB transcription factor MaEFM-like [58] and the association of nuclear localization protein FolSvp1 with tomato PR1 [24]. Therefore, further characterization of FonCHRD-interacting partners in plants and exploring their interactions and effects on plant immune responses will provide novel insight into the molecular mechanisms of FonCHRD in *Fon* pathogenicity.

Most of the effector proteins in *F. oxysporum*, such as FolAsp, FoRnt2, FSE1, FoCupin1, and FoSSP17, are irrelevant to the basic biological processes of the pathogens [10, 30]. This is primarily because these effectors are usually located on pathogenic chromosomes, such as the *SIX* genes in *Fol*, and are specifically expressed upon host plant perception during the early stage of infection [15, 59]. Surprisingly, we found that *FonCHRD* plays a critical role in basic biological processes, including vegetative growth, asexual reproduction, and macroconidial morphology (Fig. 3). This is similar to the candidate effector protein Cep28 in *F. oxysporum* f. sp. *cepae*, which is related to vegetative growth, pigmentation and virulence and has a functional tradeoff between virulence and vegetative growth [60]. Another, CHRD has been demonstrated to play crucial roles in numerous important developmental processes acts in vertebrates [35–39]. In fungi, there is a single gene encoding for CHRD domain-containing protein (Fig. 1B). Therefore, it is likely that the sole CHRD domain-containing protein, like FonCHRD, might have diverse functions in fungi. Unlike Cep28 that is essential for abiotic stress responses in *F. oxysporum* [60], FonCHRD seems not involved in cell wall, oxidative and salt stress responses in *Fon* (Fig. 4). Generally, most of the secreted proteins and effectors are crucial for pathogenicity [10]. For instance, Fosp9, a candidate effector protein, is necessary for the full virulence of *Foc* on bananas [61], and candidate effector Foc1324 has been discovered as a virulence factor required for the pathogenicity of *Foc* [62]. Three early root colonization secreted proteins, which were identified in the extracellular fluid of infected tomato roots, are essential for the full virulence of *Fol* [9]. Our studies revealed that FonCHRD is essential for *Fon* pathogenicity (Fig. 2A–C). The significant decreases in fungal biomass of Δ*FonCHRD* in roots and stems of the infected watermelon plants (Fig. 2F and G) indicated that FonCHRD may play a role in regulating invasive growth and colonization within its host plants. Furthermore, the greater decrease of fungal biomass in stems than in roots (Fig. 2F and G) also implied the impaired spreading ability of Δ*FonCHRD* from root to stem in watermelon plants. Therefore, the reduced pathogenicity of the Δ*FonCHRD* is primarily due to defects in invasive growth and spreading within watermelon plant (Fig. 2D–G), rather than a defect in penetration ability (Fig. 2H). These features resemble the functions of previously identified pathogenicity factors, including FonNst2, FonPAT2, FonPUF1, and FonPARP1, which were found to regulate *Fon* pathogenicity through affecting the invasive growth and colonization within host plants [63–66]. Thus, regulating the invasive growth and spreading abilities, rather than the penetration capacity, is one of the mechanisms by which FonCHRD contributes to *Fon* pathogenicity. However, it cannot be ruled out that the reduced pathogenicity of Δ*FonCHRD* might be attributed, at least partially, to the defects in the fundamental biological characteristics of *Fon*, e.g., vegetative growth and asexual reproduction, due to the dysfunction of *FonCHRD*.

Upon pathogen infection, rapid PCD at infection sites is crucial for plants to inhibit pathogen growth [67]. In *Agrobacterium*-mediated transient expression for analyzing the abilities of fungal effector proteins to induce or inhibit PCD [68], BAX, a pro-apoptotic protein in mouse cells, and INF1, an elicitor of *Phytophthora infestans*, triggers typical PCD, similar to plant resistance gene-mediated hypersensitive response [46, 47]. In this study, we observed that FonCHRD does not induce cell death but can inhibit BAX- or INF1-triggered PCD as well as upregulation of defense gene expression in *N. benthamiana* leaves (Figs. 6 and 7). This is consistent with the findings that FocCupin1, FocM35_1, FolAsp, and FoSSP17 suppress PCD and inhibit immune responses in plants [21, 23, 29, 30]. Notably, both SP-containing FonCHRD and SP-deleted FonCHRD suppressed the INF1- and BAX-triggered PCD in *N. benthamiana* leaves (Fig. 6). These observations indicate that the SP does not contribute to the ability of FonCHRD to inhibit INF1- and BAX-triggered PCD; instead, it has a function in directing FonCHRD to target the apoplast space in plants. This implies that localization in the apoplast space is not essential for the specific function of FonCHRD in inhibiting INF1- and BAX-triggered PCD and it is thus likely that FonCHRD exerts its function inside the plant cells to inhibit PCD. Similar observations were reported for FoSSP17 and FoCupin1, which were able to inhibit BAX-triggered PCD in SP-independent manner [29, 30]. It is plausible that FonCHRD may function in a similar manner to the previously reported effectors that suppress plant immune responses during *Fon* infection. Therefore, suppressing immune responses in host plants may be another mechanism for FonCHRD to regulate *Fon* pathogenicity for infection.

In summary, we characterized a CHRD domain-containing secreted protein FonCHRD and investigated its biological functions in *Fon*. FonCHRD comprises a N-terminal SP with secretion activity and can target to apoplast space in plants. Phenotypic analysis using the targeted deletion mutant revealed that FonCHRD plays critical roles in basic biological processes and pathogenicity of *Fon*. We also demonstrated that FonCHRD regulates *Fon* pathogenicity by affecting colonization and spreading abilities of *Fon* within watermelon plants and by suppressing plant immune responses. Future investigations on characterizing the biochemical function of FonCHRD and identifying the FonCHRD-interacting partners in both *Fon* and host plants will elucidate the molecular mechanisms by which FonCHRD, a secretory effector protein, regulates *Fon* pathogenicity, especially its interfering with the plant immune responses.

## Materials and methods

### Fungal strains, plants, and growth conditions

*Fon* race 1 strain ZJ1 was used as the wild type (WT) strain for generating deletion mutants and phenotypic analyses. *Fon* strains were grown at 26℃ on PDA or MM plates [64]. Conidiation and spore germination assays were performed using MBL and yeast extract peptone dextrose (YEPD) broth at 26 °C, respectively [63]. Watermelon (*Citrullus lanatus* L. cv. Zaojia) used for the disease assays were grown in a potting mix (vermiculite: plant ash: perlite = 6:2:1) in a growth room at 22 ~ 24 °C with a 14-h light/10-h dark cycle [65]. *N. benthamiana* plants used for subcellular localization and transient expression were grown in the potting mix in a growth room at 22 °C with a 16-h light/8-h dark cycle [69].

### Bioinformatics analysis and effector prediction

CHRD domain, transmembrane domain, and GPI anchor sites were scanned using the online SMART (http://smart.embl-heidelberg.de/), TMHMM 2.0 (http://www.cbs.dtu.dk/services/TM-HMM-2.0/), and big-PI Fungal Predictor (https://mendel.imp.ac.at/gpi/fungi_serve-r.html) tools, respectively [70, 71]. Signal peptide was identified by SignalP-5.0 (www.cbs.dtu.dk/services/SignalP), while subcellular localization was predicted using ProtComp 9.0 (http://linux1.softberr-y.com) and WoLF PSORT (www.wolfpso-rt.org) [52, 72]. Effector values of FonCHRD were determined by EffectorP 3.0 (https://effectorp.csiro.au/) [29]. The secondary and tertiary structures of FonCHRD were modeled using SOPMA (https://npsapbil.ibcp.fr/cgibin/npsa_aut-omat.pl?pag-e=npsa_sopma.html) and SWISS-MODEL (https://swissmodel.expas-y.org/) tools. Multiple sequence alignment and phylogenetic tree construction were performed using the Clustalw and MEGA5.0 programs, respectively.

### Assays for the secretion activity of SP

The SP sequence in *FonCHRD* was inserted into pSUC2 vector, which contains a SUC2 truncated invertase lacking its own SP and start codon [44], at *EcoR*I and *Xho*I sites, yielding pSUC2-FonCHRD^SP^. The recombinant pSUC2-FonCHRD^SP^, pSUC2-Avr1b^SP^ [45], and empty pSUC2 were separately transformed into the invertase secretion-deficient yeast strain YTK12 using lithium acetate-mediated transformation, and the transformants were screened on CMD-W medium (SD/-Trp medium; Clontech, Mountain View, CA, USA). Yeast strains carrying pSUC2-FonCHRD^SP^, pSUC2-Avr1b^SP^ or pSUC2 were grown on YPRAA (1% yeast extract, 2% peptone, 2% raffinose, 2 μg antimycin A, and 2% agar in 1 L distilled H_2_O) plates with antimycin A (2 μg/mL). After incubation in 10% sucrose solution at 37℃ for 10 min, the secretion activity of SPs in different yeast strains were estimated by adding 0.1% 2,3,5-triphenyltetrazolium chloride (TTC) solution for coloration [44].

### Generation of the targeted deletion mutants and complementation strains

To construct the targeted deletion vector, two 0.5 ~ 1.0 kb fragments flanking *FonCHRD* were amplified and fused with the hygromycin resistance gene (*HPH*) fragment through double-joint PCR method [73]. The purified PCR products were introduced into *Fon* through protoplast transformation [63]. Positive transformants were selected on PDA containing 100 μg/mL hygromycin B and verified through PCR and Southern blotting, leading to the identification of three deletion mutant lines Δ*FonCHRD-7*, Δ*FonCHRD-11*, and Δ*FonCHRD-20*. To construct the complementation vector, a genomic fragment including the entire *FonCHRD* coding sequence together with its native promoter was amplified and inserted into *Xho*I-digested pYF11-neo plasmid by co-transforming into yeast strain XK-125 [66]. The complementation vector was then transformed into Δ*FonCHRD* through protoplast transformation. Positive transformants were selected on PDA supplemented with 50 μg/mL G418 and confirmed by PCR amplification of the transgene and Western blotting detection of the FonCHRD-GFP fusion with an anti-GFP antibody [64].

### Pathogenicity assays and fungal biomass estimation

Pathogenicity assays were performed as previously described [63]. Roots of three-week-old watermelon plants were submerged in *Fon* spore suspension (5 × 10^6^ spores/mL) or MBL as mock controls for 15 min and were then replanted in soil. The inoculated plants were covered with plastic wrap for 3 d to provoke the disease development. Disease symptom was recorded using a 4-scale rating standard (0 = no symptoms, 1 = chlorosis, 2 = wilting, and 3 = death) and survival rate was calculated every three days. For tissue examination of fungal colonization, roots and stems were collected from the inoculated plants at 15 dpi, sterilized with ethanol, and incubated on PDA for 3 d [63]. For assessment of fungal biomass, the roots of three-week-old watermelon plants were submerged in *Fon* spore suspension and incubated at 26℃ with shaking (95 rpm). Roots and stems were sampled at 3, 6, and 9 dpi. Relative fungal biomass, expressed as *FonOpm12*/*ClRps10* ratio, was determined using a quantitative polymerase chain reaction (qPCR)-based quantification method as previously described [66]. The penetration ability of *Fon* strains was determined as previously described [74]. Briefly, *Fon* strains were initially cultivated on MM plates covered with cellophane for 3 d and kept inoculation for another 2 d after the cellophane was removed.

### Phenotypic analysis of basic biological traits and stress response assays

The basic biological phenotypes of the *Fon* strains were examined following previously described methods [66, 74]. For assessment of vegetative growth, the colony diameters of *Fon* strains grown on PDA or MM for 7 d at 26 °C were recorded. In conidiation assays, different *Fon* strains were cultivated in MBL broth at 26 °C in a shaker (250 rpm) for 2 d and macroconidia were counted using a hemocytometer [74]. In spore germination assays, macroconidia from different *Fon* strains were incubated in YEPD broth at 26 °C with shaking (250 rpm) for 12 h and the germination of macroconidia were microscopically examined as described previously [74]. Macroconidia from different *Fon* strains were stained with 10 μg/mL calcofluor white. Their morphology and septation were observed under a confocal microscope [63]. For stress response assays, different *Fon* strains were cultivated on PDA containing 0.7 M NaCl (Sinopharm Chemical, Shanghai, China), 0.7 M CaCl_2_ (Sinopharm Chemical), 0.1% paraquat (Syngenta Crop Protection, Basel, Switzerland), 0.1% H_2_O_2_ (Sigma-Aldrich, St. Louis, MO, USA), 0.2 g/L CR (Sigma-Aldrich) or 0.3 g/L SDS (Sigma-Aldrich) and incubated at 26 °C for 7 d. The mycelial growth inhibition rate was calculated as described previously [63].

### Transient expression and cell death suppression assays

The coding sequence of *FonCHRD* and the fragment for SP-deleted variant *FonCHRD*^ΔSP^ were amplified from *Fon* cDNA. The fragment for NbPR1 SP was fused to *FonCHRD*^ΔSP^ using double-joint PCR to generate *FonCHRD*^NbPR1SP^. The *FonCHRD*, *FonCHRD*^ΔSP^, and *FonCHRD*^NbPR1SP^ fragments were then cloned into pGR107 vector at *Cla*I and *Not*I sites using T4 DNA Ligase (TaKaRa, Kusatsu, Japan) for cell death assays [21, 48] or into pCAMBIA1300 vector at *Kpn*I rsites using ClonExpress II One Step Cloning Kit (Vazyme Biotech, Nanjing, China) for subcellular localization assays. Primers used are listed in Table S1.

The recombinant vectors carrying the tested genes were transformed into *Agrobacterium tumefaciens* strain GV3101. Positive transformants were cultured in Luria–Bertani broth containing 50 μg/mg kanamycin and 25 μg/mg rifampicin and incubated at 28℃ in a shaker (220 rpm) to OD_600_ = 0.8. After centrifugation, agrobacteria were suspended in infection solution (10 mM MgCl_2_, 5 mM MES and 150 μM acetosyringone) and adjusted the concentration to OD_600_ = 1.0. After incubation at 28℃ for 3 h, the bacterial suspension was injected into the back of four-week-old *N. benthamiana* leaves. For cell death suppression assays, *N. benthamiana* leaves were first injected agrobacteria carrying the test genes at 24 h before injecting agrobacteria harboring BAX or INF1 [21, 30]. Cell death phenotype was recorded at 4 d post-agroinfiltration, and the dead cells were detected with Trypan blue staining [48].

### Subcellular localization assay

Agrobacteria carrying pCAMBIA1300‐FonCHRD‐GFP, pCAMBIA1300‐FonCHRD^ΔSP^‐GFP, pCAMBIA1300‐FonCHRD^NbPR1SP^‐GFP or empty vector pCAMBIA1300 were infiltrated into RFP‐H2B *N. benthamiana* leaves and fluorescent signal was examined under a Zeiss LSM780 confocal microscope (Gottingen, Niedersachsen, Germany) at 48 h after infiltration [69]. To examine the targeting in apoplast space, the agroinfiltrated leaves were incubated in 0.8 M mannitol solution for 0.5 h to induce plasmolysis, and fluorescent signal was observed under the confocal microscope.

### Protein extraction and Western blotting

Samples of fungal mycelia and *N. benthamiana* leaf tissues were ground into fine powders in liquid nitrogen and mixed in a protein extraction buffer (1 M Tris–HCl, pH7.4, 0.5 M EDTA, 1 M NaCl, 0.2% Triton X-100, 1 mM DTT, and 1 × protease inhibitor cocktail). Total proteins were extracted as previously described [63] and separated on 12.5% SDS-PAGE gels, followed by transferring onto an Immobilon-P transfer membrane (Millipore, Billerica, MA, United States). Proteins on the membranes were immunoblotted with anti-GFP antibody (Cat. #ab290, Abcam, Cambridge, United Kingdom), anti-GAPDH antibody (Cat. #EM1101, HuaBio, Hangzhou, China) or anti-HA antibody (Cat. #sc-7392, Santa Cruz Biotechnology, Shanghai, China) and detected using the ECL chromogenic reagent (Cat. #36208ES60, Yeasen Biotechnology, Shanghai, China). The immunoblotted membranes were photographed using Tanon automatic gel imaging system (Tianneng Corporation, Shanghai, China).

### Sampling, RNA extraction, qPCR, and RT‑qPCR assays

For analysis of *FonCHRD* expression, *Fon* was grown in 50 mL YEPD broth with or without 1 g watermelon roots at 26℃, 200 rpm, and mycelia were collected at different time points after incubation. *Fon* was cultured in 50 mL MBL broth and macroconidia were collected. Macroconidia were germinated in 50 mL YEPD broth and harvested at 26℃, 200 rpm for 12 h. Agroinfiltrated *N. benthamiana* leaves were collected at 48 h after agroinfiltration.

All the samples were ground into fine powders in liquid nitrogen and total RNA was extracted using RNA Isolater Reagent (Vazyme, Nanjing, China). First-strand cDNA was reversely transcribed from 1 μg total RNA using HiScript II qRT SuperMix kit (Vazyme, Nanjing, China). qPCR reactions were prepared using 2 × AceQ qPCR SYBR Green Master Mix (Vazyme, Nanjing, China) with gene-specific primers (Table S1) and run on a LightCycler 96 instrument (Roche, Basel, Switzerland). *FonActin* and *NbGAPDH* were used as internal controls to normalize the qPCR data for *FonCHRD* in *Fon* and defense genes in *N. benthamiana*, respectively. Relative expression of genes was calculated using the 2^−ΔΔCT^ method [75].

### Statistical analysis

All the experiments were conducted independently three times. Statistical analysis was performed by analysis of variance (ANOVA) or unpaired Student’s *t*-test and significant difference was defined by probability values of *p* < 0.05 or *p* < 0.01.

## Supplementary Information

Supplementary Material 1. Fig. S1. Characterization of FonCHRD and its protein structure. Fig. S2. Generation and characterization of the targeted deletion mutant Δ*FonCHRD* and the complementation strain Δ*FonCHRD*-C. Fig. S3. Different deletion mutant strains of Δ*FonCHRD* exhibited similar phenotype with decreased pathogenicity on watermelon plants. Fig. S4. Subcellular localization of FonCHRD in *N. benthamiana* leaves. Table S1. Primers used in this study.

Supplementary Material 2.

## Data Availability

The authors confirm that the data supporting the findings of this study are available within the article and its supplementary materials.

## Abbreviations

BAX: Bcl2-associated X protein
CHRD: Chordin
CR: Congo Red
dpi: Days post inoculation
*Foc*: *Fusarium oxysporum* F. sp. *cubense*
*Fol*: *Fusarium. oxysporum* F. sp. *lycopersici*
*Fon*: *Fusarium oxysporum* F. sp. *niveum*
FonCHRD: *Fusarium oxysporum* F. sp. *niveum* CHRD
GFP: Green fluorescent protein
GPI: Glycosylphosphatidylinositol
HA: Hemagglutinin
MBL: Mung bean liquid
MM: Minimal medium
NbPR1: *Nicotiana benthamiana* Pathogenesis-related protein 1
PDA: Potato dextrose agar
PCD: Programmed cell death
qPCR: Quantitative polymerase chain reaction
RT-qPCR: Reverse transcription-quantitative polymerase chain reaction
SDS: Sodium dodecyl sulfate
SP: Signal peptide
YEPD: Yeast extract peptone dextrose
YPRAA: Yeast extract-peptone-raffinose-antimycin A-agar
WT: Wild-type
