# Supplementary material for: The secreted protein FonCHRD is essential for vegetative growth, asexual reproduction, and pathogenicity in watermelon Fusarium wilt fungus
Source: Crop Health. 2024 Oct 25;2(1):16. doi: 10.1007/s44297-024-00036-x (PMC12825985; doi:10.1007/s44297-024-00036-x)
Supplement: Supplementary file 1 — Supplementary Material 1. Fig. S1. Characterization of FonCHRD and its protein structure. Fig. S2. Generation and characterization of the targeted deletion mutant ΔFonCHRD and the complementation strain ΔFonCHRD-C. Fig. S3. Different deletion mutant strains of ΔFonCHRD exhibited similar phenotype with decreased pathogenicity on watermelon plants. Fig. S4. Subcellular localization of FonCHRD in N. benthamiana leaves. Table S1. Primers used in this study. [file 44297_2024_36_MOESM1_ESM.docx]

**Supplemental Materials**

**The secreted protein FonCHRD is essential for vegetative growth, asexual reproduction, and pathogenicity in watermelon Fusarium wilt fungus**

Jiajun Lou ^a,b,c^, Jiajing Wang ^a,b,c^, Shanshan Zeng ^a,b,c^, Xiaohui Xiong ^a,b,c^, Mengmeng Guo ^a,b,c^, Dayong Li ^a,b,c^, and Fengming Song ^a,b,c,*^

^a^ Key Laboratory of Crop Diseases and Insect Pests of Ministry of Agriculture and Rural Affairs, Institute of Biotechnology, College of Agriculture and Biotechnology, Zhejiang University, Hangzhou 310058, People’s Republic of China

^b^ Zhejiang Provincial Key Laboratory of Biology of Crop Pathogens and Insects, Institute of Biotechnology, College of Agriculture and Biotechnology, Zhejiang University, Hangzhou 310058, People’s Republic of China

^c^ State Key Laboratory of Rice Biology and Breeding, Institute of Biotechnology, College of Agriculture and Biotechnology, Zhejiang University, Hangzhou 310058, People’s Republic of China

*Correspondence:

Fengming Song

fmsong@zju.edu.cn


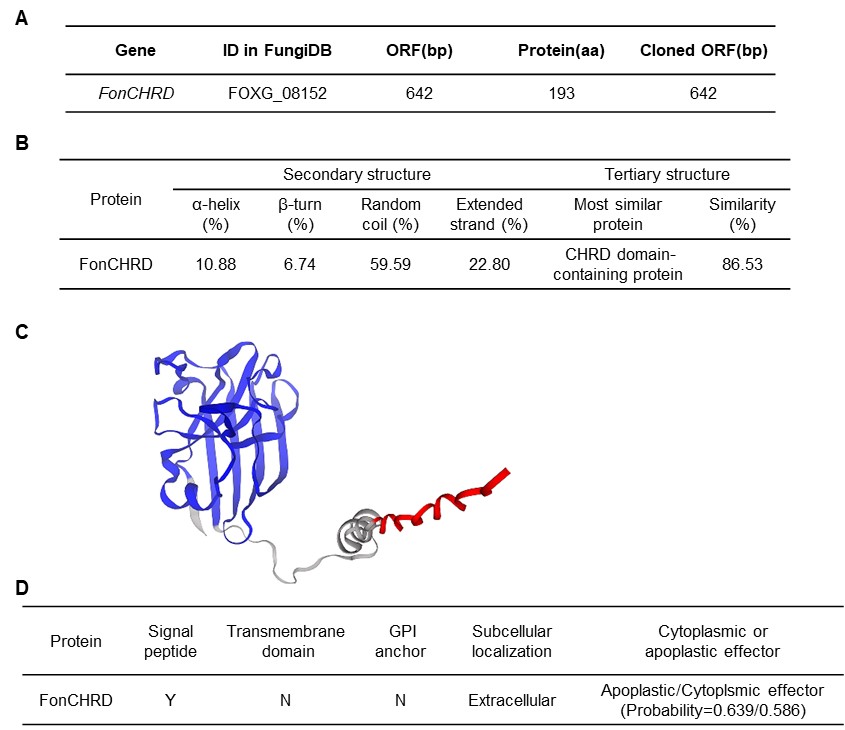


**Fig. S1** Characterization of FonCHRD and its protein structure.

**A** Information on FonCHRD gene and its protein. **B** Information on secondary and tertiary structures of FonCHRD. **C** Predicted three-dimensional structure of FonCHRD. **D** Characteristics of FonCHRD as a candidate effector.


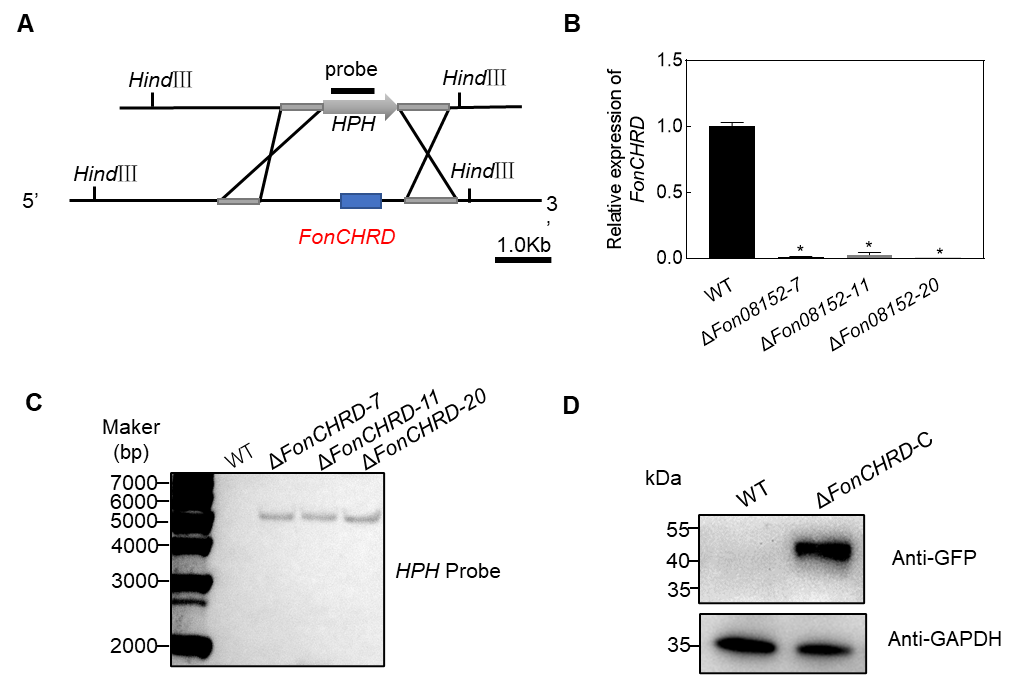


**Fig. S2** Generation and characterization of targeted deletion mutant Δ*FonCHRD* and complementation strain Δ*FonCHRD*-C.

**A** A schematic diagram showing the construction of deletion vector for FonCHRD. The HPH fragment used as a probe is indicated. **B** Relative transcript level of *FonCHRD* in WT and Δ*FonCHRD*. Total RNA was extracted from 2-d-old cultures of WT or mutant strains and reversely transcribed into cDNAs used for RT-qPCR. *FonActin* was used as an internal reference. **C** Southern blot analysis of Δ*FonCHRD* using the HPH probe. **D** Western blot detection of FonCHRD-GFP in the complementation strain Δ*FonCHRD*-C. Data presented in (**B**) are means ± SD from three independent experiments, and asterisks indicate significant differences (*p* < 0.05, Student’s *t* test).


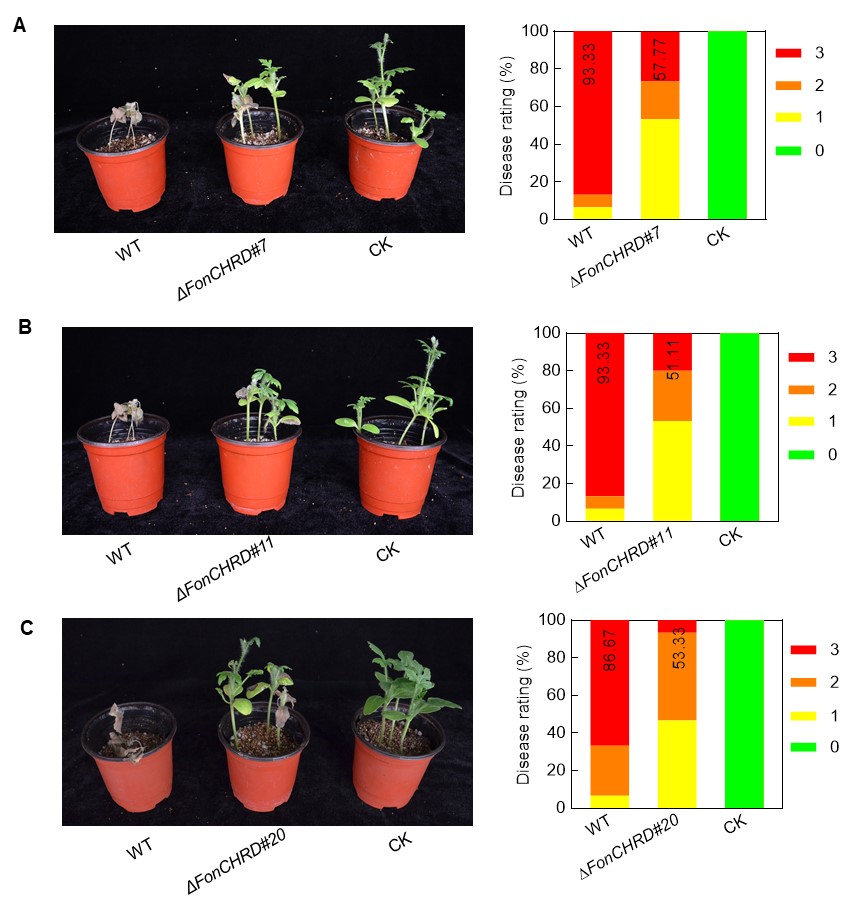


**Fig. S3** Different deletion mutant strains of Δ*FonCHRD* exhibited similar phenotype with decreased pathogenicity on watermelon plants.

**A**–**C** Disease phenotype (*left*) and disease index (*right*) of watermelon plants after inoculation with or WT strains. Photographs were taken at 21 dpi. The experiments were independently conducted three times with similar results and data are means ± SD from three independent experiments.


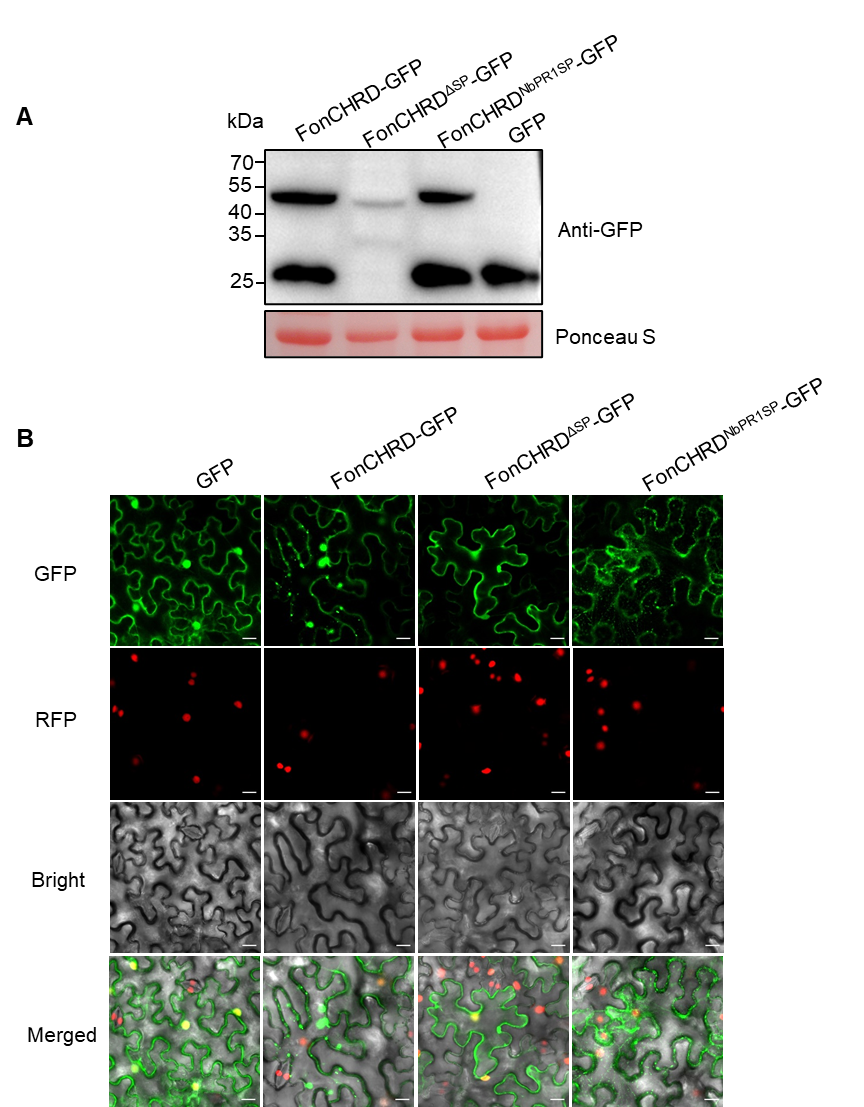


**Fig. S4** Subcellular localization of FonCHRD in *N. benthamiana* leaves.

**A** Protein levels of FonCHRD-GFP, FonCHRD^ΔSP^-GFP and FonCHRD^NbPR1SP^-GFP in transiently expressed *N. benthamiana* leaves. Ponceau S staining was used for equal loading of protein samples. **B** Subcellular localization of FonCHRD-GFP, FonCHRD^ΔSP^-GFP and FonCHRD^NbPR1SP^-GFP in transiently expressed *N. benthamiana* leaves. Bars = 20 μm. Photographs were taken after 48 h post-agroinfiltration. Experiments were independently performed three times with similar results and data from one representative experiment are shown.

**Table S1** Primers used in this study

| **Primers** | **Sequences (5’-3’)** |
| --- | --- |
| ***Generation and characterization of deletion mutant and complementation strains*** | |
| FonCHRD-5’-F | CGGATTGACCCAGGATCAGA |
| FonCHRD-5’-R | CAAAATAGGCATTGATGTGTTGACCTCCCGACGCCGAC  ATTCACCAT |
| FonCHRD-3’-F | CGTCCGAGGGCAAAGGAATAGAGTAGTCCTCGGGGTT  GACGACTGT |
| FonCHRD-3’-R | CCTGGATCTTTGACGATGGTTG |
| FonCHRD-YZ1-F | CAGGCACCTTGGAAAGAATACG |
| FonCHRD-YZ1-R | AGTCACATACGGTCAGGGCATC |
| FonCHRD-YZ2-F | ATGAAATTCTCTCTCTTTGC |
| FonCHRD-YZ2-R | GCACACCTTGCCTGAGCTGAG |
| HPH-F | GGAGGTCAACACATCAATGCCTATT |
| HPH-R | CTACTCTATTCCTTTGCCCT |
| HPH-probe-F | CACGATAACTTGGTGCGTTTG |
| HPH-probe-R | TCCAGTCAATGACCGCTGTTA |
| G418-neo-F | GGAGGTCAACACATCAATGCT |
| G418-neo-R | TCAGAAGAACTCGTCAAGAAG |
| G418YZ-F | AAGATGGATTGCACGCAGGTT |
| G418YZ-R | AAGAAGGCGATAGAAGGCGAT |
| FonCHRD-PYF11-F | ACTCACTATAGGGCGAATTGGGTACTCAAATTGGTTCA  GAACCGAATGACCCCACC |
| FonCHRD-PYF11-R | CACCACCCCGGTGAACAGCTCCTCGCCCTTGCTCACG  CACACCTTGCCTGAGCTGAG |
| ***Assays for signal peptide activity*** | |
| FonCHRDSP-pSUC2-F | CCCGAATTCATGAAATTCTCTCTCTTTGCCG |
| FonCHRDSP-pSUC2-R | GGGCTCGAGGGCAGCAGCGAGGGTGCT |
| ***Assays for subcellular localization and transient expression*** | |
| PGR107-F | TCTCAAGCCACTCTCCGTT |
| PGR107-R | TGAGGTAGTTGACCCTATGG |
| NbPR1SP-ClaI-F | CCCATCGATATGAATTTTACTGGCTATTC |
| NbPR1SP-1300-F | AGCTTTCGCGAGCTCGGTACCATGAATTTTACTGGCTATTC |
| FonCHRD-ClaI-F | CCCATCGATATGAAATTCTCTCTCTTTGCCG |
| FonCHRD-NotI-R | GGGGCGGCCGCGCACACCTTGCCTGAGCTGAG |
| FonCHRD^△SP^-ClaI-F | CCCATCGATATGTCGCCAACTCCTGACATGG |
| FonCHRD-NbPR1SP-F | ATGAATTTTACTGGCTATTCTCGATTTTTAATCGTCTTTG  TAGCTCTTGTAGGTGCTCTTGTTCTTCCCTCGAAAGCTTC  GCCAACTCCTGACATGG |
| FonCHRD-1300-F | AGCTTTCGCGAGCTCGGTACCATGAAATTCTCTCTCTTTGCCG |
| FonCHRD-1300-R | GCCCTTGCTCACCATGGTACCGCACACCTTGCCTGAGCTG |
| FonCHRD^△SP^-1300-F | AGCTTTCGCGAGCTCGGTACCATGTCGCCAACTCCTGACATGG |
| ***RT-qPCR assays*** | |
| FonActin-RT-F | GAGGGACCGCTCTCGTCGT |
| FonActin-RT-R | GGAGATCCAGACTGCCGCTCAG |
| FonOpm12-RT-F | CGATTAGCGAAGACATTCACAAGACT |
| FonOpm12-RT-R | ACGGTCAAGAAGATGCAGGGTAAAGGT |
| ClRps10-RT-F | AGGCTCACCCTAAAAGAAGG |
| ClRps10-RT-R | GGTCAACACAAGGATCTTACT |
| FonCHRD-RT-F | CTCTCTTTGCCGTTGCTCTC |
| FonCHRD-RT-R | CAGGGGTAGCAAGAATTTCCCA |
| ClGAPDH-RT-F | ATGGGCAAAGTTAAGATCGGCATCA |
| ClGAPDH-RT-R | CCAATTCGATATCATCACTCTGC |
| ClPR1-RT-F | CTTGAGCTTTGCCATGCTGC |
| ClPR1-RT-R | GCGTTGGTTGGCATATTGTCG |
| ClPR5-RT-F | CCAGCAACATTAACGAGCGG |
| ClPR5-RT-R | ACTCGACCAGTGTATGGTGC |
| ClPR2-RT-F | CCGTCAACTGTGGTCACTGA |
| ClPR2-RT-R | CTCCGCCAACCTTCTCCAAT |
| ClEDS1-RT-F | GAGCAAGCAGGCATATGGGA |
| ClEDS1-RT-R | GCTCAATGAGACGGCGAAAC |
| ClEBF1-RT-F | ACCATGGAGGATTGGGGAAG |
| ClEBF1-RT-R | TCGCCGATGGATGCAACATT |
| ClAOC-RT-F | AGCTTTCAGCCACCTCCATC |
| ClAOC-RT-R | TCTGGGTTTGAGAGAGGGGT |
| ClLOX-RT-F | ACCCCGTAGTTATTGCTCGT |
| ClLOX-RT-R | GCCTCATCCACAGTTAGCCC |
| ClERF1a-RT-F | AATTGGGGCGATTTGCCTCT |
| ClERF1a-RT-R | CCTCCGGCTCTGATTTCACC |
| ClEIN2-RT-F | GCTTTGCACACCTTTCCAGA |
| ClEIN2-RT-R | GTCAGGACCAGTTGACCACC |
| ClEIN3-RT-F | TCTTCCCTCAGTCGTTTGCC |
| ClEIN3-RT-R | GCCATCTTCTGGAACGCCTA |
| NbPR1a-q PCR-F | CCGCCTTCCCTCAACTCAAC |
| NbPR1a-q PCR-R | GCACAACCAAGACGTACTGAG |
| NbPR2-q PCR-F | AGGTGTTTGCTATGGAATGC |
| NbPR2-q PCR-R | TCTGTACCCACCATCTTGC |
| NbLOX-q PCR-F | AAAACCTATGCCTCAAGAAC |
| NbLOX-q PCR-R | ACTGCTGCATAGGCTTTGG |
| NbERF1-q PCR-F | GCTCTTAACGTCGGATGGTC |
| NbERF1-q PCR-R | AGCCAAACCCTAGCTCCATT |
| NbHIN1-q PCR-F | CCAACTTGAACGGAGCCTATTA |
| NbHIN1-q PCR-R | AGGCATCCAAAGAGACAACTAC |
| NbHSR203J-q PCR-F | ACGCAGATTTCAACCGAGTAT |
| NbHSR203J-q PCR-R | GCCAGTCGCATTGGAGATAA |
| NbGAPDH-RT-F | AGCTCAAGGGAATTCTCGATG |
| NbGAPDH-RT-R | AACCTTAACCATGTCATCTCCC |
