# Supplementary material for: The secreted protein FonCHRD is essential for vegetative growth, asexual reproduction, and pathogenicity in watermelon Fusarium wilt fungus
Source: Crop Health. 2024 Oct 25;2(1):16. doi: 10.1007/s44297-024-00036-x (PMC12825985; doi:10.1007/s44297-024-00036-x)

Photos for uncropped blots

**FonCHRD, a CHRD domain-containing secreted protein with cell death inhibitory activity, is essential for vegetative growth, asexual reproduction, and pathogenicity in watermelon Fusarium wilt fungus**

Jiajun Lou ^a,b,c^, Jiajing Wang ^a,b,c^, Shanshan Zeng ^a,b,c^, Xiaohui Xiong ^a,b,c^, Mengmeng Guo ^a,b,c^, Dayong Li ^a,b,c^, and Fengming Song ^a,b,c,*^

^a^ Key Laboratory of Crop Diseases and Insect Pests of Ministry of Agriculture and Rural Affairs, Institute of Biotechnology, College of Agriculture and Biotechnology, Zhejiang University, Hangzhou 310058, People’s Republic of China

^b^ Zhejiang Provincial Key Laboratory of Biology of Crop Pathogens and Insects, Institute of Biotechnology, College of Agriculture and Biotechnology, Zhejiang University, Hangzhou 310058, People’s Republic of China

^c^ State Key Laboratory of Rice Biology and Breeding, Institute of Biotechnology, College of Agriculture and Biotechnology, Zhejiang University, Hangzhou 310058, People’s Republic of China

*Correspondence:

Fengming Song

fmsong@zju.edu.cn

**Fig. 6B**

Anti-GFP


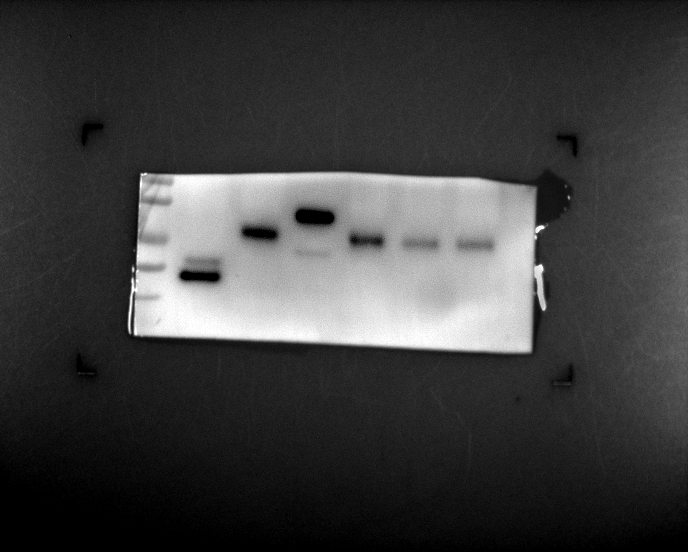


Ponceau S


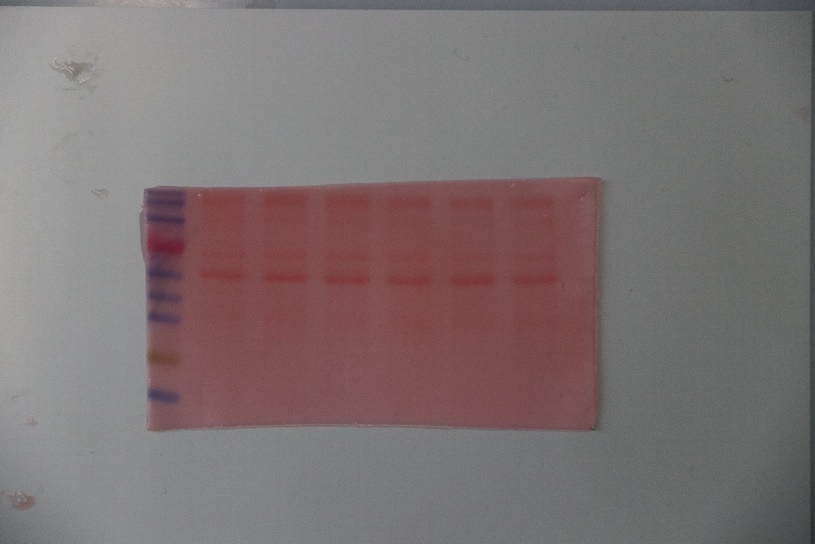


**Fig. 6D**

Anti-GFP, left lanes 1-5


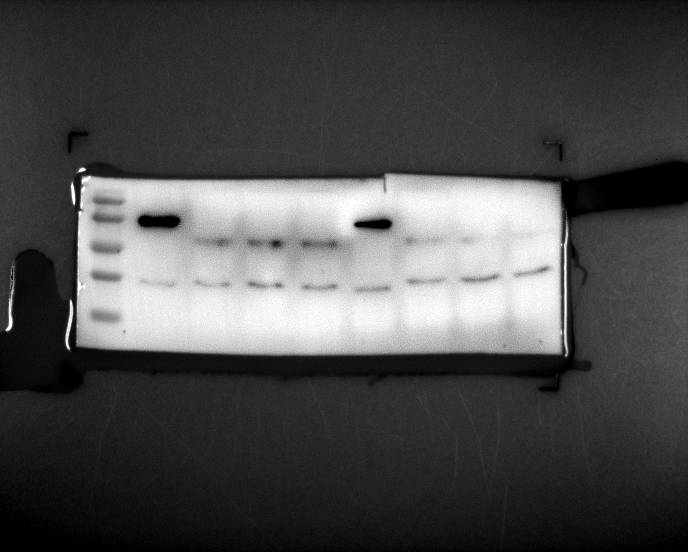


Ponceau S


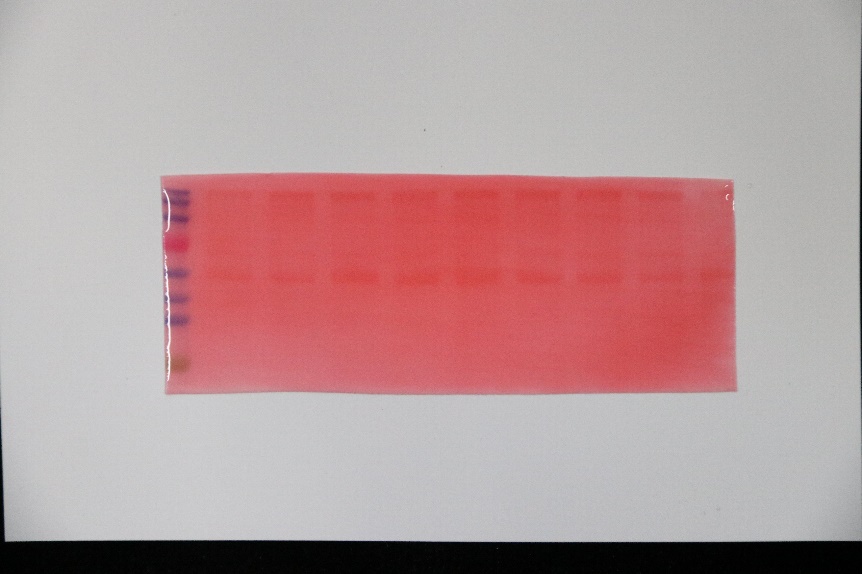


**Fig. 6E**

Anti-GFP, left lanes 1-5


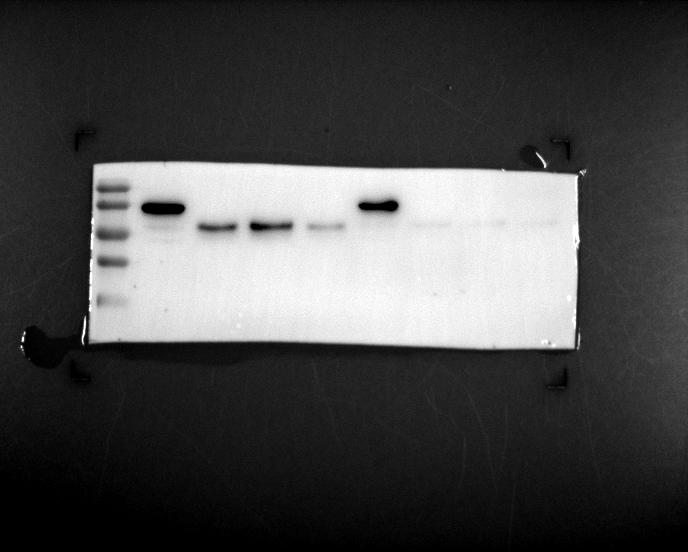


Ponceau S


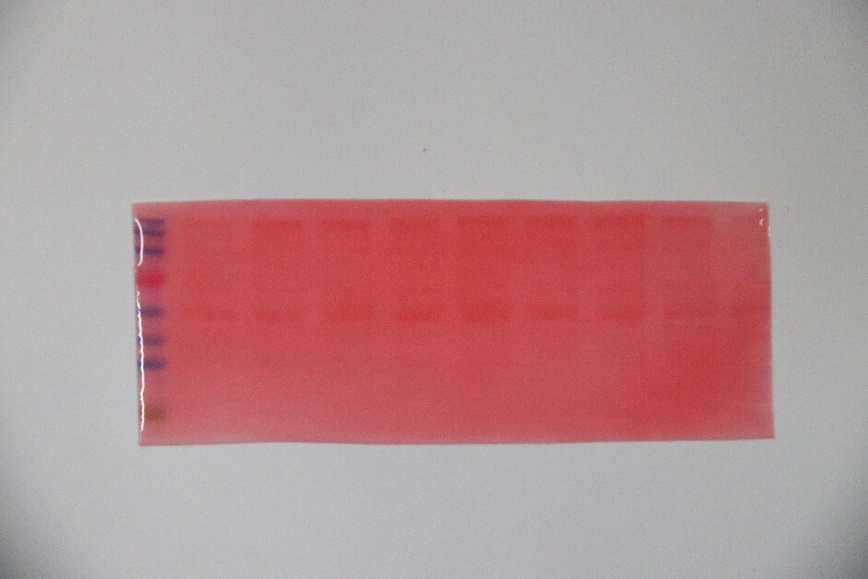


**Fig. S2C**

left lanes 1-5


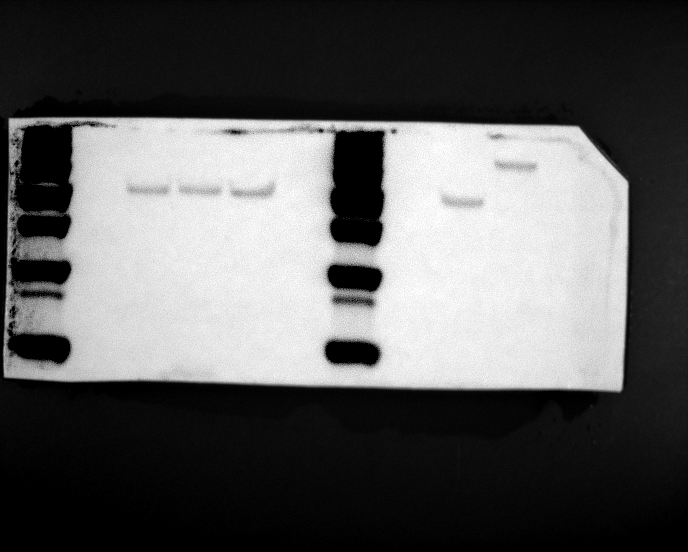


**Fig. S2D**

Anti-GFP, left lanes 2-3 (lane 1 is protein marker)


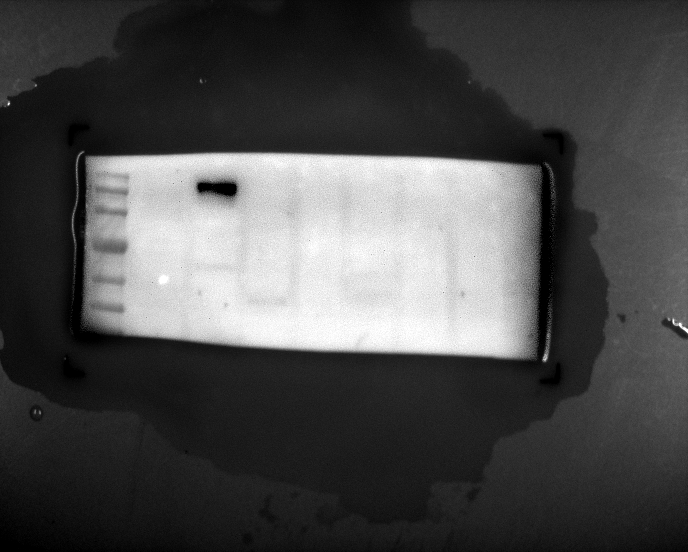


Anti-GAPDH, left lane 2-3


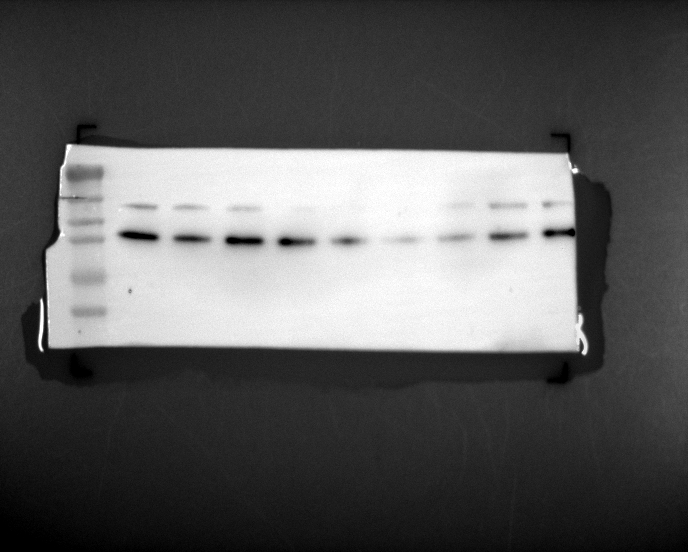


**Fig. S3A**

Anti-GFP


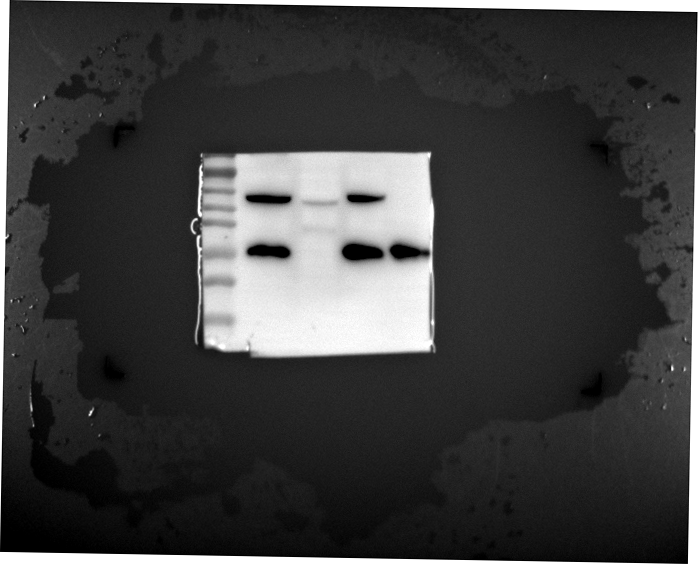


Ponceau S


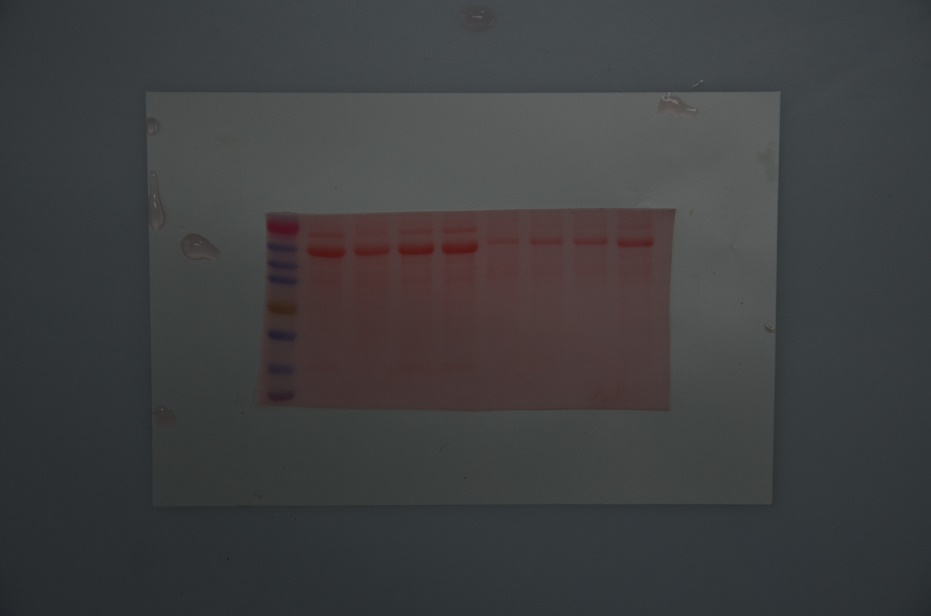

Supplement: Supplementary file 2 — Supplementary Material 2. [file 44297_2024_36_MOESM2_ESM.docx]
